# COVID-19 vaccine acceptance among health care workers in Africa: A systematic review and meta-analysis

**DOI:** 10.1371/journal.pone.0268711

**Published:** 2022-05-18

**Authors:** Martin Ackah, Louise Ameyaw, Mohammed Gazali Salifu, Delali Pearl Afi Asubonteng, Cynthia Osei Yeboah, Eugene Narkotey Annor, Eunice Abena Kwartemaa Ankapong, Hosea Boakye

**Affiliations:** 1 Department of Physiotherapy, Korle Bu Teaching Hospital, Accra, Ghana; 2 Department of Epidemiology, School of Public Health, College of Health Sciences, University of Ghana, Accra, Ghana; 3 Department of Medicine, Achimota Hospital, Accra, Ghana; 4 Policy Planning Budgeting Monitoring and Evaluation Directorate, Ministry of Health, Accra, Ghana; 5 Department of Physiotherapy, GA East Municipal Hospital, Accra, Ghana; 6 School of Public Health, East Tennessee State University, Johnson City, Tennessee, United States of America; 7 Department of Occupational Therapy, School of Biomedical and Allied Health Science, University of Ghana, Accra, Ghana; 8 Department of Physiotherapy, LEKMA Hospital, Accra, Ghana; Xiamen University - Malaysia Campus: Xiamen University - Malaysia, MALAYSIA

## Abstract

**Introduction:**

Coronavirus Disease (COVID-19) vaccine acceptance, and hesitancy amongst Health Care Workers (HCWs) on the African continent have been examined through observational studies. However, there are currently no comprehensive reviews among these cadre of population in Africa. Hence, we aimed to review the acceptance rate and possible reasons for COVID-19 vaccine non-acceptance/hesitancy amongst HCWs in Africa.

**Methods:**

We searched Medline/PubMed, Google Scholar, and Africa Journal Online from January, 2020 to September, 2021. The Newcastle-Ottawa Quality Assessment tool adapted for cross-sectional studies was used to assess the quality of the retrieved studies. DerSimonian and Laird random-effects model was used to pool the COVID-19 vaccine acceptance rate. Sub-group and sensitivity analyses were performed. Reasons for COVID-19 vaccine hesitancy were also systematically analyzed.

**Results:**

Twenty-one (21) studies were found to be eligible for review out of the 513 initial records. The estimated pooled COVID-19 vaccine acceptance rate was 46% [95% CI: 37%-54%]. The pooled estimated COVID-19 vaccine acceptance rate was 37% [95% CI: 27%-47%] in North Africa, 28% [95% CI: 20%-36%] in Central Africa, 48% [CI: 38%-58%] in West Africa, 49% [95% CI: 30%-69%] in East Africa, and 90% [CI: 85%-96%] in Southern Africa. The estimated pooled vaccine acceptance was 48% [95% CI:38%-57%] for healthcare workers, and 34% [95% CI:29%-39%] for the healthcare students. Major drivers and reasons were the side effects of the vaccine, vaccine’s safety, efficacy and effectiveness, short duration of the clinical trials, COVID-19 infections, limited information, and social trust.

**Conclusion:**

The data revealed generally low acceptance of the vaccine amongst HCWs across Africa. The side effects of the vaccine, vaccine’s safety, efficacy and effectiveness, short duration of the clinical trials, COVID-19 infections, limited information, and social trust were the major reasons for COVID-19 hesitancy in Africa. The misconceptions and barriers to COVID-19 vaccine acceptance amongst HCWs must be addressed as soon as possible in the continent to boost COVID-19 vaccination rates in Africa.

## Introduction

The current Coronavirus Disease (COVID-19) pandemic is a global public health emergency that offers significant challenges to health-care systems [1, 2]. ‘‘Coronaviruses are large, enveloped, positive-strand RNA viruses that can be categorized into genera; alpha, beta, delta and gamma, of which alpha and beta are known to infect humans” [3]. Human Corona Viruses (HCoVs) i.e. HCoV 229E, NL63, OC43 and HKU1 are endemic globally and account for 10%- 30% of upper respiratory tract infections in adults humans [3].

The current basic reproductive number (R0) of Severe Acute Respiratory Syndrome Coronavirus-2 (SARS COV-2) is estimated to be three and as a result the threshold of herd immunity for COVID-19 is roughly around 67 percent [4, 5]. This purport that after the population’s acquired immunity reaches 67 percent and above, COVID-19 infection rates will start to decline [6].

Individual and community initiatives such as enhanced hand cleanliness, physical distancing, and the personal protective equipment are currently being used to reduce disease transmission. However, with the world facing an economic downturn and an uncertain future, a COVID-19 vaccine is perhaps the best option for halting the epidemic [7, 8].

The SARS Cov-2 Development and Access Strategy established by Africa Center for Disease Control in 2020 aim to vaccinate at least 60% of African Population by 2022 to develop herd immunity [9]. Africa has received approximately 143 million doses in total as of September, 2021, but only 39 million people, or around 3% of the continent’s population, had been adequately vaccinated. In the United States, 52 percent of people are fully vaccinated, whereas in the European Union, 57 percent are [10]. The willingness of Health Care Workers (HCWs) to be vaccinated against COVID-19 acts as a valuable role model for the general public [11].

As the vaccine becomes more widely available in Africa, Sevidzem et al identified and evaluated some probable link to vaccination acceptability in Africa. The factors included vaccination deployment plans, religious practices, vaccine hesitation, proliferation of misinformation, HCW attitudes towards the vaccine, social effects, and supportive environment [12]. Vaccine aversion among the general public has a direct association to vaccine hesitancy among HCWs [13]. Thus, HCWs role in vaccine acceptability cannot be underestimated as a result of their modeling behavior [13].

A rapid systematic review of global vaccine acceptance among HCWs ranged from approximately 28% to 73% [6]. Similarly, a comprehensive review and meta-analysis of cross-sectional studies of health workers’ intentions to vaccinate against COVID-19 indicated a moderate acceptance rate [i.e., 51 percent]. The authors did admit, however, that the population studied were largely from economically developed countries, which limited the study’s generalizability [14]. Clearly, this cannot be extended to represent HCW intentions to vaccinate against COVID-19 in Africa.

COVID-19 vaccine acceptance, and hesitancy amongst HCWs on the African continent have been examined through observational studies [7, 15]. However, there are currently no comprehensive reviews among these cadre of population in Africa. Hence, we aimed to systematically review the acceptance rate and possible reasons for COVID-19 Vaccine non-acceptance/hesitancy amongst HCWs in Africa. The outcome would enable stakeholders [i.e., policy makers, researchers and government] package effective health promotion measures to boost COVID-19 vaccine uptake in Africa.

### Specific objectives

1. To determine the level of COVID-19 vaccine acceptance among HCWs in Africa.
2. To assess the drivers of COVID-19 vaccine non-acceptance/hesitancy among HCWs in Africa.

## Methods

### Protocol registration and best practice

The Center for Reviews and Dissemination standards were followed in preparing this systematic review and meta-analysis [16]. Also, the current review was conducted and reported according to the guidelines of the Preferred Reporting Items for Systematic Review and Meta-Analysis (PRISMA) [17] [see S1 Table]. The protocol was registered at PROSPERO: [CRD42021275065].

### Eligibility criteria

#### Inclusion criteria

1. HCWs, and health science students from Africa continent, were included. HCW was operationally defined as; Doctors, Nurses, Pharmacists, allied health professionals, paramedics, and Healthcare students [i.e., medical students, nurse students etc.].
2. Adults HCWs aged ≥18 years were included.
3. All primary studies such as longitudinal, cohort, case-control and cross-sectional studies reporting COVID-19 vaccine acceptance and hesitancy among HCWs in Africa were included in the current review.
4. Original observational studies published in English were included.

#### Exclusion criteria

1. General population, other university students, and children were excluded.
2. Non-COVID-19 vaccine acceptance studies.
3. COVID-19 studies reporting animal studies, reviews, commentaries, letter to editors were excluded.
4. COVID-19 vaccine acceptance and hesitancy articles published in other language other than English were excluded
5. COVID-19 acceptability studies among HCWs outside the Africa continent [i.e., Asia, Europe, America, and Australia continents] were also excluded.

### Outcome of interest

The outcome of interest was COVID-19 vaccine acceptance/and hesitancy rate among HCWs in Africa. In addition, the reasons for COVID-19 hesitancy were explored.

### Information sources and search strategies

Medline/PubMed, Google Scholar, Africa Journal Online, and MedRxiv (preprint) were searched. The search was restricted to studies published between January,2020 to September, 2021. The search was limited to articles published in English. Reference lists of articles that met the inclusion and exclusion criteria were reviewed manually to identify additional studies.

Medical Sub-Heading (MeSH) terms and free text were used in the search approach. These terms were combined with the Boolean operators ‘OR’ and ‘AND’. The key terms included; COVID-19, Vaccine, Hesitancy, acceptance, Health care worker, Africa, Sub-Saharan Africa. The full search string is shown in S2 Table.

### Data screening and selection

The data screening and selection involved the following; Two co-authors independently screened the titles and abstracts against the eligibility criteria. Full texts of the articles were then obtained. A disagreement was then resolved by consensus. To ensure that independent reviewers apply the selection criteria reliably, a screening guide was used [18].

### Data extraction and management

Two co-authors extracted the data from the eligible published articles using a pre-tested and standardized excel spreadsheet. Data such as the author’s name, year of publication, country, survey period, study design, sample size, HCWs population, acceptance rate, and, reasons for COVID-19 acceptance/hesitancy rate were extracted. Mendeley was used to managed and remove duplicated articles.

### Quality assessment and risk of bias

The Newcastle-Ottawa Quality Assessment tool adapted for cross-sectional studies [19] was used to assess the quality of the retrieved studies. It is graded on 10-point stars. This process was done by two independent reviewers and average was taken as a final score for that particular study. The Newcastle-Ottawa Quality Assessment tool contains three domains. Domain 1 evaluates the methodological quality of each study [5 stars], domain 2 assesses the comparability of the study [2 stars] and domain 3 evaluates the outcome measure and related statistical analysis [3 stars] [19]. Furthermore, the review rated the overall quality of the studies into three; [low risk of bias (7–10), moderate risk of bias (5–6), and high risk of bias (0–4)] [20].

### Data synthesis

Extracted data was exported into Stata (version 16; Stata Cooperation, TX, USA) from Microsoft excel 2013 for all analyses. Due to the presence of heterogeneity [I^2^ = 96%, p≤0.001], a meta-analysis using the random effect model was used to pool the COVID-19 vaccine acceptance rate among the HCWs at 95% confidence interval and presented in a forest plot. The presence of heterogeneity among studies was quantified by estimating the variance using the I^2^ statistics [21]. The I^2^ takes values between 0 and 100%, and a value of 0% indicates absence of heterogeneity. I^2^ was interpreted based on Higgins and Thompson classification, percentages of 25%, 50% and 75% was considered as low, moderate and high heterogeneity, respectively [21]. A sub-group analysis was performed based on sub-region (West Africa vs. East Africa vs. Southern Africa vs. North Africa) and type of participants (Healthcare workers vs. Healthcare students). Leave one out sensitivity analysis was performed to examine the effects of a single study on the overall pooled estimate. Publication bias was checked by the funnel plot and Egger’s test. The drivers/factors for COVID-19 vaccine non-acceptance/hesitancy among HCWs in Africa were systematically reviewed. A factor/driver for COVID-19 vaccine non-acceptance/hesitancy was eligible if it had been assessed and data from at least two studies were available.

## Results

**Fig 1:**
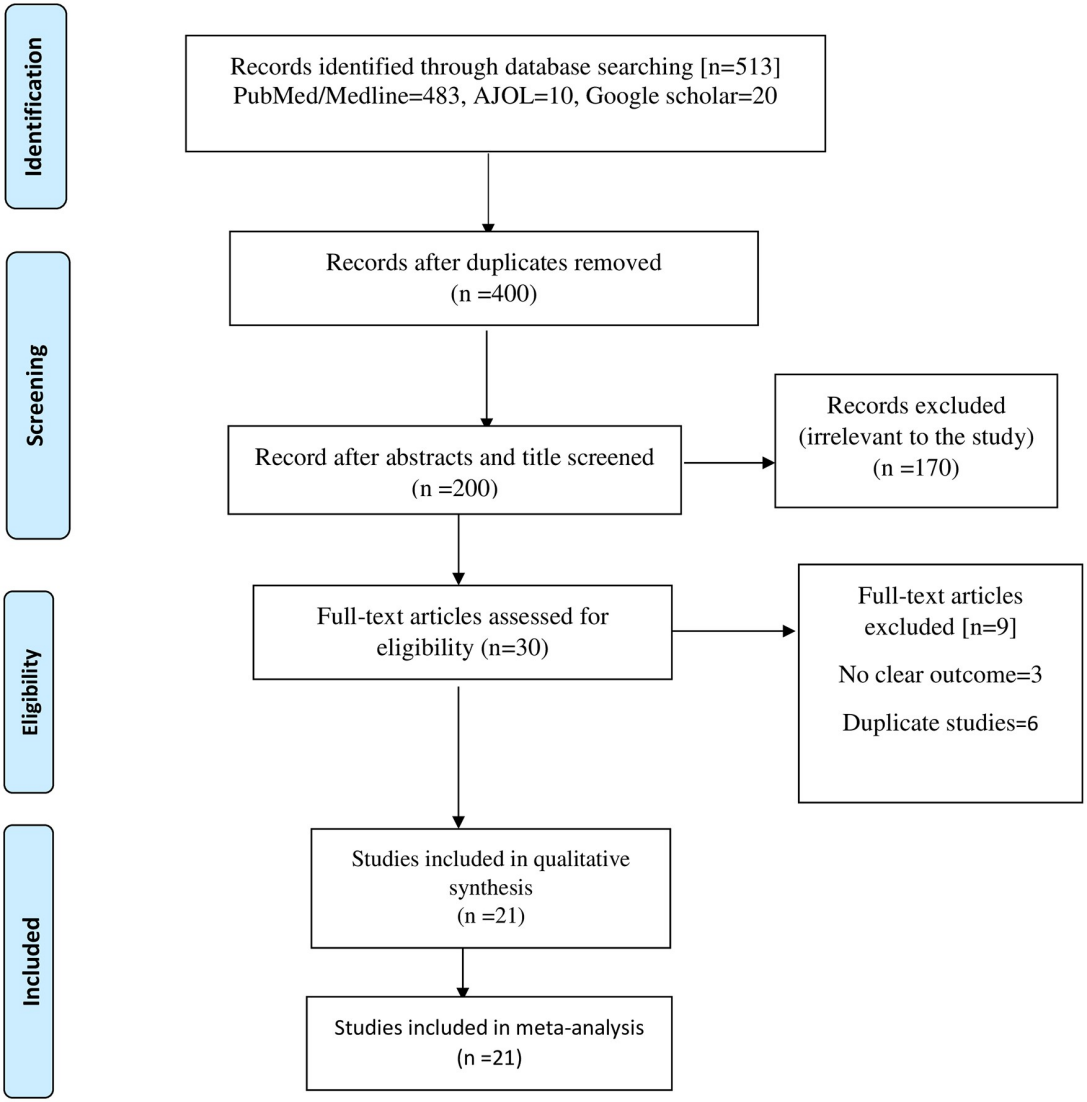
Preferred Reporting Items for Systematic Review and Meta-Analysis-Adapted flow showing the results of the search.

The electronic search yielded 513 articles; 400 articles remained after the duplicate articles were deleted. After screening the abstracts and titles, 200 articles were removed [i.e., irrelevant to the study]. One hundred and seventy (170) were removed because they were unrelated to the current research. Thirty (30) full-text papers were evaluated for eligibility. Nine papers were removed from the final data synthesis, leaving only 21 articles. The results are displayed in Fig 1.

### Characteristics of the studies

Out of the 21 studies included, 7 were conducted in North Africa, 6 in West Africa, 6 in East Africa and an article each from central and southern Africa. The sample size ranged from 182 to 2133, totaling 14132 participants. The participants were mainly doctors, nurses, medical laboratory scientists, pharmacists, and allied health staff. The first survey was performed in March-April 2020, and the most recent was conducted in March-June 2021. The studies were all cross sectional and published between 2020 and 2021. Majority of the included studies had low-moderate risk of bias [20/21]. The findings are summarized in Table 1.

**Table 1:** Characteristics of the studies [n = 14132].

Author and Year	Country	Participants	Survey period	Male (%)	Age/Years	Sample Size	Acceptance rate n (%)	Reasons for Vaccine Hesitancy	Quality assessment
Nzaji, 2020 [22]	DR Congo	Doctors, Nurses, Midwives, and Laboratory Technicians	March/April, 2020	50.9	40.31±11.67, Majority; 25–40 (63%)	613	27.7	Not stated	Low
Fares, 2021 [23]	Egypt	Doctors, Nurses, Pharmacists, Physiotherapists, and Dentists	Dec, 2020-Jan, 2021	18.7	Majority;17–35 (70.4%)	385	21	Lack of enough clinical trials, and Fear of vaccine’s side effects	Low
El-Sokkary, 2021 [24]	Egypt	Doctors, Dentists, Pharmacist, and others	Jan, 2021	22.4	NA	308	26	Severity of COVID-19 Vaccine safety	Moderate
Agyekum, 2021 [15]	Ghana	Doctors, Nurses/midwives, and Allied health	Jan/Feb, 2021	36.8	Majority; 30–39 (56.0%)	234	39.3	vaccine safety vaccine side effects Acquiring COVID-19 through vaccination	Moderate
Dula, 2021 [7]	Mozambique		March, 2021	NA	NA	566	86.6	Vaccine side effects Made to cause harm Vaccine not effective	Moderate
Adeniyi, 2021 [25]	South Africa	Doctors, Nurses, Pharmacists, Allied Health, Support staff	Nov/Dec, 2020	18.5	Majority; 26–55 (79.2%)	1308	90.1	Not stated	Low
Shehata, 2021 [26]	Egypt	Doctors	March/June, 2021	40.6	Majority: 31–40 (71.5%)	1268	24.3	Vaccine side effects Short duration of Clinical Trial Concerns about safety and efficacy	High
Saied,2021 [27]	Egypt	Healthcare Students	Jan, 2021	34.8	20.2±1.8	2133	34.9	Insufficient information about vaccine side effect Insufficient information about the vaccine Insufficient trust from vaccine source	Moderate
Kanyike, 2021 [28]	Uganda	Healthcare Students	March, 2021	62.8	Majority: <25 (61.2%)	600	37.3	Vaccine side effects Misinformation ineffectiveness	Low
Ngasa, 2021 [29]	Cameroon	Doctor, Nurse, laboratory technician, Pharmacist, Public health, student, Other	Not stated	51.8	29.1±6.6	371	45.4	Efficacy of the vaccine Short duration of clinical trials Adverse effects	Low
Aliae, 2021 [30]	Egypt	Doctor, Nurse, laboratory technician, Pharmacist, student, Other	Dec, 2020-Jan, 2021	34.9	Majority; 18–45 (55.0%)	496	45.9	Not stated	Moderate
Alle,2021 [31]	Ethiopia	Doctor, Anesthetists, Nurses, Midwives, Pharmacists, Laboratory Professional	Not stated	63.6	Majority; 18–25 (55.0%)	327	42.3	Not stated	Low
Guangul, 2021 [32]	Ethiopia	Physicians, health officers, nurses, Lab Technicians, Pharmacist, others	Not stated	69.3	Majority; 18–29 (58.2%)	668	72.2	Concerns about safety, Ineffective Acquiring COVID-19 through vaccination Side effects, Short duration of clinical trial	Low
Ahmed, 2021 [33]	Ethiopia	All Health professionals	Jan-March, 2021	70.2	Majority;30–39 (54.0%)	409	33.2	Not stated	Low
Annan, 2021 [34]	Ghana	Doctors	Not stated	49.2	Majority; 25–30 (83.0%)	305	66.9	Not stated	Low
Adejumo,2021 [35]	Nigeria	Doctors, Nurse, Lab scientist, pharmacist, Physiotherapist, others	Oct, 2020	64.3	40.0±6.0, Majority 18–40 (72.9%)	1470	55.5	Not stated	Low
Robinson,2021 [36]	Nigeria	All Health professionals	Dec, 2020-Jan, 2021	56.7	Majority; 30–49 (66.6%)	1094	48.8	Safety, Ineffectiveness Side effects Fear of the unknown	Moderate
Oriji, 2021 [37]	Nigeria	Nurses, Lab scientist, Pharmacist, others	April, 2021	25.3	Majority; <36 (47.8)	182	27.4	To see what will happen (fear) Short duration of Clinical trial Side effects Safety issues Lack of trust in government/manufacturer	Moderate
Khairy, 2021 [38]	Sudan	Doctors, Nurse, Lab scientist, pharmacist, public health, others	March- April 2021,	46.7	35.3±10.6	576	57	Not stated	Low
Zammit, 2021 [39]	Tunisia	Doctors, Nurses pharmacy, paramedic	Jan, 2021	26.6	37.4 ±9.5, Majority; <41 (66.1%)	493	48.1	Not stated	Low
Mudenda,2021 [40]	Zambia	Pharmacy student	April, 2021	50.3	Majority; 18–29 (81.2%)	326	24.5	Side effects Ineffectiveness Safety issues Short clinical trials	Moderate

#### Pooled COVID-19 vaccine acceptance rate among HCWs in Africa

**Fig 2:**
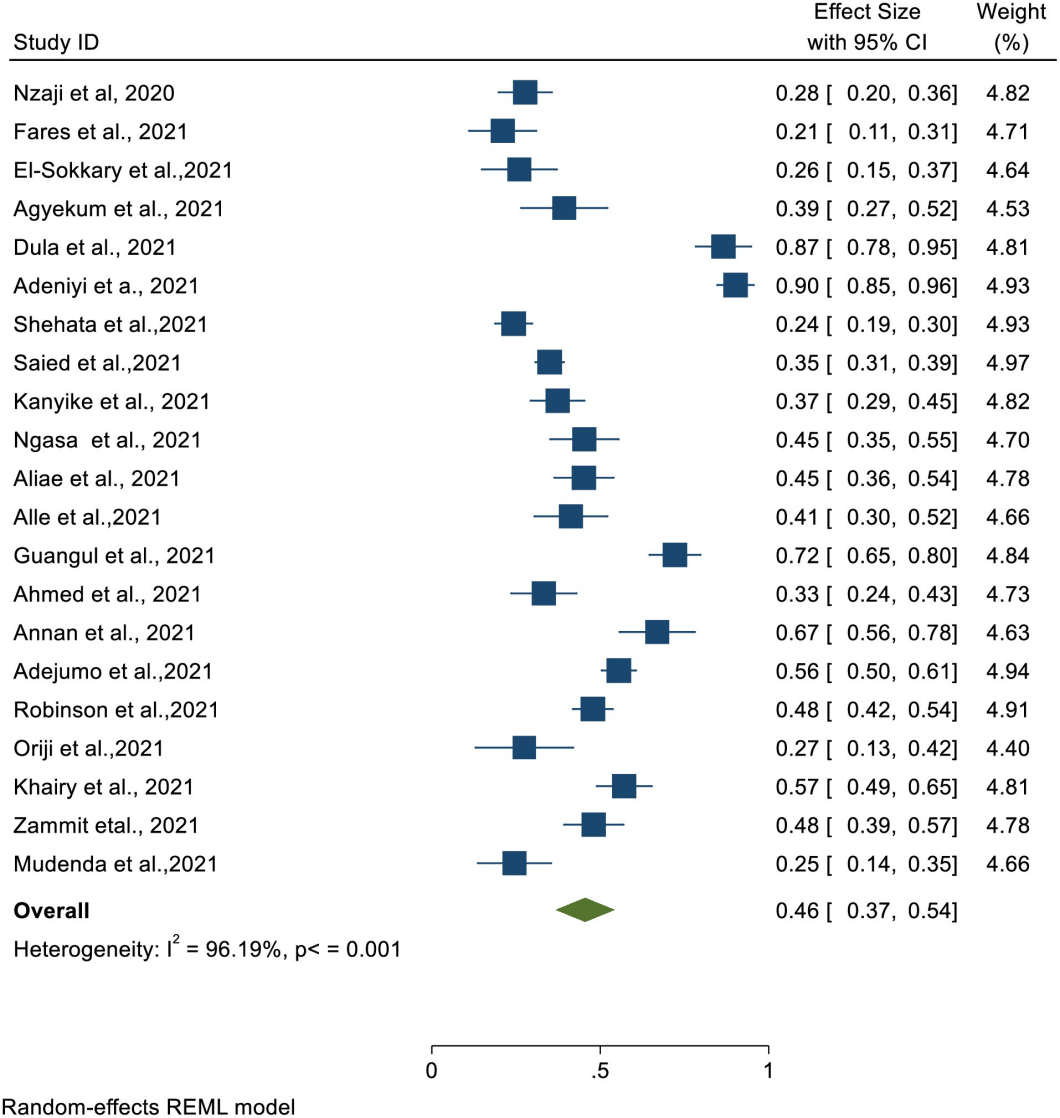
COVID -19 vaccine acceptance rate among HCWs in Africa.

The COVID-19 vaccination acceptance rate was calculated using data from twenty-one (21) studies in Africa. Based on the DerSimonian and Laird random-effects model, meta-analysis revealed a pooled COVID-19 acceptance rate of 46% [95% CI: 37%-54%] (Fig 2). However, there was significant variability among the studies [I^2^ = 96%, p≤0.001].

### Publication bias assessment

**Fig 3:**
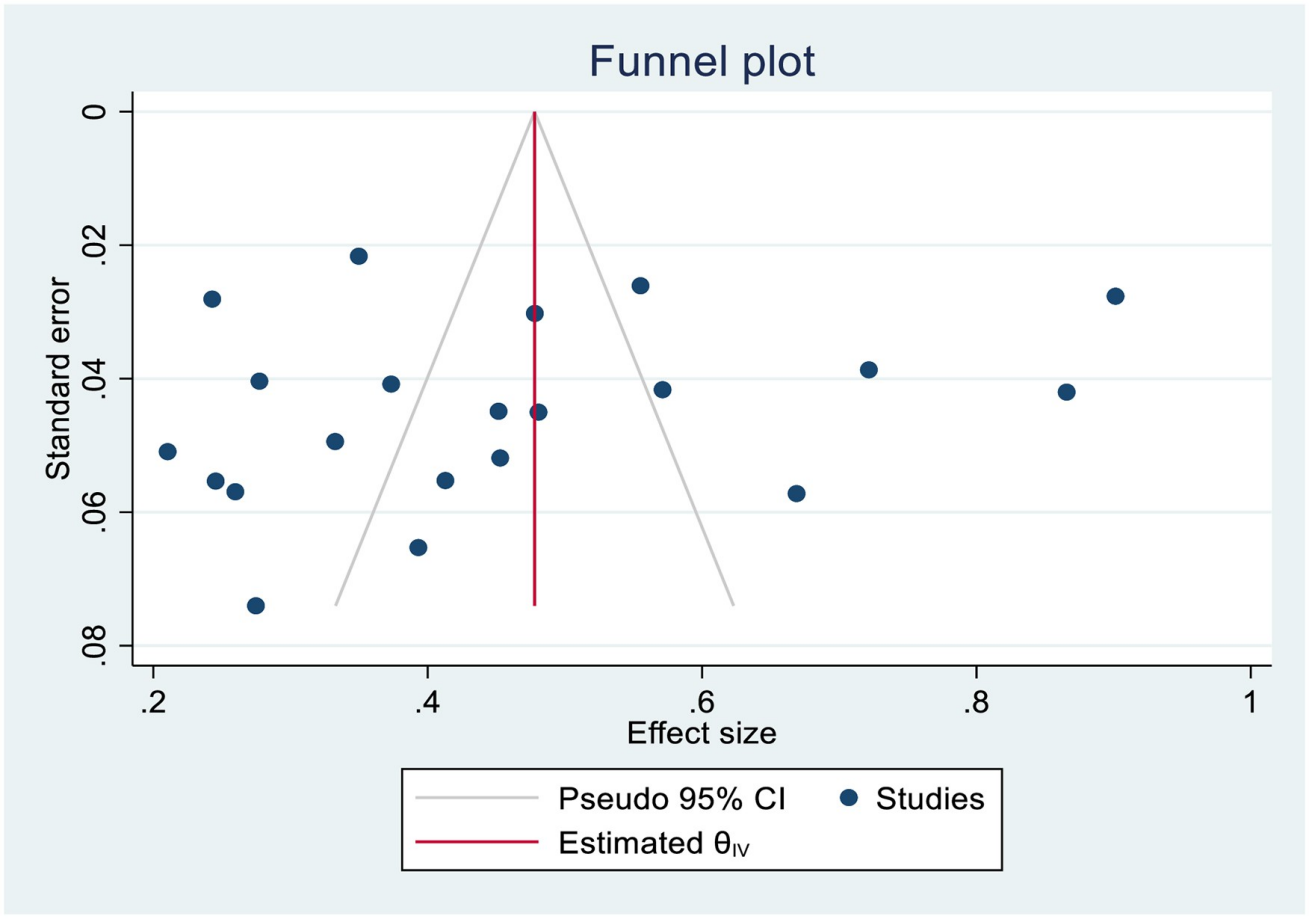
Assessment of publication bias.

No evidence of publication bias was found after symmetrical inspection using the funnel plot (Fig 3) and Egger’s regression test (0.1654).

### Sub-group and sensitivity analysis for COVID-19 vaccine acceptance rate

#### Sub-group analysis

As shown in Table 2, sub-group analysis was based on sub-regions (i.e., North Africa vs. Central Africa, East Africa vs. West Africa). The pooled estimated COVID-19 acceptance rate was 37% [95% CI: 27%-47%] in North Africa, 28% [95% CI: 20%-36%] in Central Africa, 48% [CI: 38%-58%] in West Africa, 49% [95% CI: 30%-69%] in East Africa, and 90% [CI: 85%-96%] in Southern Africa.

**Table 2:** Pooled COVID-19 vaccine acceptance rate stratified by sub-region and type of participants.

Group	Number of datasets	Pooled estimate at 95%CI	I^2^ [p-value]
**Sub-region**			
Central Africa	1	28% [95% CI: 20%-36%]	-
North Africa	7	37% [CI: 27%-47%]	92.47% [p≤0.001]
West Africa	6	48% [CI: 38%-58%]	87.31% [≤0.001]
South Africa	1	90% [CI: 85%-96%]	-
East Africa	6	49% [95% CI: 30%-69%]	96.50% [≤0.001]
**Type of participants**			
Health workers	18	48% [95% CI: 38%-57%]	96.14% [≤0.001]
Health science student	3	34% [95% CI: 29%-39%]	37.13% [≤0.001]

CI = Confidence Interval

Similarly, further stratification by type of participants (health workers vs. health science students). The estimated pooled COVID-19 vaccine acceptance was 48% [95% CI: 38%-57%] for HCWs, and 34% [95% CI: 29%-39%] for the healthcare students [Table 3].

**Table 3:** Reasons for COVID-19 vaccine hesitancy among HCWs in Africa.

Reason	Number of studies	References
Side effects of the vaccine	10	[7, 15, 23, 26, 28, 29, 32, 36, 37, 40]
Vaccine’s safety	7	[15, 23, 24, 26, 36, 37, 40]
Efficacy and effectiveness	7	[7, 26, 28, 29, 32, 36, 40]
Short duration of the clinical trials	6	[23, 26, 29, 32, 37, 40]
COVID-19 infections	2	[15, 32]
Limited information	2	[27, 28]
Lack of Social trust	2	[27, 37]

#### Sensitivity analysis for COVID-19 acceptance rate

Leave one out sensitivity analysis was performed to re-estimate the pooled effect on outcome of the remaining studies on the pooled COVID-19 acceptance rate. The results revealed that, no single study had a signifcant impact on the overall result. The pooled acceptability rate of COVID-19 vaccine ranged from 43% [95% CI: 35%-52%] to 47% [95% CI: 38%-55%] (S3 Table).

### Reasons for COVID-19 vaccine non-acceptance among HCWs in Africa

The current systematic review and meta-analysis identified 8 main reasons for COVID-19 vaccine hesitancy among HCWs in Africa. These includes: the side effects of the vaccine [7, 15, 23, 26, 28, 29, 32, 36, 37, 40] the vaccine’s safety [15, 23, 24, 26, 36, 37, 40], efficacy and effectiveness [7, 26, 28, 29, 32, 36, 40], short duration of the clinical trials [23, 26, 29, 32, 37, 40], COVID-19 infections [15, 32], limited information [27, 28], and lack of social trust [insufficient trust in the vaccine’s source, lack of trust from the manufacturers, lack of trust from governments] [27, 37]. The results are summarized in Table 3.

## Discussion

The systematic review, and meta-analysis was carried out to ascertain the COVID-19 vaccine acceptance rate, and possible reasons for the vaccine’s hesitancy amongst HCWs in Africa. The data revealed generally low acceptance of the vaccine amongst HCWs across Africa, and considerable COVID-19 vaccine reluctance. The possible reasons for the vaccine’s hesitancy were: the side effects of the vaccine, concerns about the vaccine’s safety, efficacy and effectiveness, short duration of the clinical trials, COVID-19 infections, limited information, and social trust.

The overall acceptance rate for the COVID-19 vaccination was 46% [95% CI: 37%-54%]. This is comparable to a previous systematic review and meta-analysis from the western world 51% [14] and higher than a US observational based study of 36% [41]. However, our estimate is lower than prior observational studies conducted in China 86.2% [42], France 76.9% [43], Saudi Arabia 64.9% [44], Canada 80.9% [45], Germany 91.7% [46] and United Kingdom 59% [47]. Low confidence in the vaccine, invasion of media misinformation, conspiracy theories, infodemic, religious beliefs, and possibly past vaccine hesitancy in the continent could all be factors contributing to the low COVID-19 vaccination acceptance rate [48, 49].

The study also revealed an estimated COVID-19 vaccine acceptance rate of 34% [95% CI:29%-39%] by the healthcare students. The estimated value is lower than previous studies in Italy 91.9% [50], US 53% [51], and France 58% [52]. Complacency, exacerbated by low illness risk, and low mortality in the continent since the epidemic began, could be contributing factors in this group of participants [53].

Certain common impediments to the acceptance of the COVID-19 vaccine seemed to be shared by HCWs across the continent. These included, side effects of vaccines, vaccine safety, efficacy and effectiveness of the vaccines, short duration of the clinical trials, the possibility of contracting COVID-19 infection from vaccines, limited information on the vaccines, and lack of social trust (i.e., insufficient trust in the vaccine’s source, lack of trust from the manufacturers, lack of trust from governments). vaccine hesitancy is mostly induced by the dissemination of misleading information, primarily through social media platforms and with the assistance of anti-vaccination organizations [54]. Biswas and colleagues conducted a scoping review analysis of 35 studies and found that HCWs worldwide have a 22.5% COVID-19 acceptance rate on average. Their reasons for refusing the vaccination were identical to those revealed in this study [11].

In general, persuading individuals who are vaccine skeptics to change their beliefs is difficult, especially in a continent where there has been a history of vaccination resistance. Nevertheless, it’s preferable to concentrate on disseminating positive and factual vaccine information while also strengthening healthcare workers’ resistance to fraudulent information. Easy access and mandatory COVID-19 vaccination policies in Africa is a good way to promote COVID-19 immunization uptake. Encouragement of vaccine production within Africa, and comparison of these vaccines with others produced outside the continent, could build more confidence in the safety and efficacy of vaccines among health care workers in the continent. This would involve isolating local strains of the virus to be used in the production of vaccines and the conduction of clinical trials among locals. The outcome of this will be, more tailored interventions to the fight against the pandemic on the continent, and will bring the fight against COVID-19 nearer home. This will also help debunk unfavorable theories about the intents behind the production of vaccines. Finally, the onus is on governments and significant international stakeholders in the pandemic fight to utilize social media to educate the public, especially HCWs, on facts concerning vaccines in order to help debunk some claims made by conspiracy theorists.

This systematic review and meta-analysis have a number of limitations that should be acknowledged. First, the current review considered only English published papers and as a result some relevant articles maybe missed. Secondary, there was significant heterogeneity across the studies. Nevertheless, to the best of the authors’ knowledge, this is the first systematic review and meta-analysis on COVID-19 acceptance and hesitancy rate in Africa. The review used well-validated systematic review and meta-analysis models that are fully compliant with current international standards and recommendations. Sensitivity analyses were performed to determine the robustness of the estimates obtained from the meta-analysis.

## Conclusion

The result of this review revealed generally low acceptance of the COVID-19 vaccine amongst HCWs across Africa. Major drivers and reasons were the side effects of the vaccine, vaccine’s safety, efficacy and effectiveness, short duration of the clinical trials, COVID-19 infections, limited information, and social trust. The willingness of HCWs to be vaccinated against COVID-19 acts as a valuable role model for the general public and hence, the misconceptions and barriers to COVID-19 vaccine acceptance amongst these cadre of professionals must be addressed as soon as possible in the continent.

## Supporting information

S1 Table PRISMA checklist. (DOC)

S2 Table Search strategy for the databases. (DOCX)

S3 Table Leave one out sensitivity analysis. (DOCX)

## References

[1] Zhou F. Clinical Course And Risk Factors For Mortality Of Adult In Patients With COVID-19 In Wuhan, China: A Retrospective Cohort Study. J Med Study Res. 2020;3(1):01–2. doi: 10.1016/S0140-6736(20)30566-3 32171076 PMC7270627

[2] Rieg S, von Cube M, Kalbhenn J, Utzolino S, Pernice K, Bechet L, et al. COVID-19 in-hospital mortality and mode of death in a dynamic and non-restricted tertiary care model in Germany. PLoS One [Internet]. 2020;15(11 November):1–16. Available from: doi: 10.1371/journal.pone.0242127 33180830 PMC7660518

[3] Paules CI. Coronavirus Infections—More Than Just the Common Cold. JAMA. 2020;323(8):707–8. doi: 10.1001/jama.2020.0757 31971553

[4] Randolph HE. Herd immunity: Understanding Covid-19 by Haley etal. Immunity. 2020;(January):19–21. 10.1016/j.immuni.2020.04.012 PMC7236739 32433946

[5] Salje H, Lefrancq N, Courtejoie N, Bosetti P, Paireau J, Andronico A, et al. Erratum: Estimating the burden of SARS-CoV-2 in France (Science 10.1126/science.abc3517). Science (80-). 2020;368(6498):208–11. 10.1126/science.abc3517 PMC7223792 32404476

[6] Li M, Luo Y, Watson R, Zheng Y, Ren J, Tang J, et al. Healthcare workers’ (HCWs) attitudes and related factors towards COVID-19 vaccination: A rapid systematic review. Postgrad Med J. 2021;52:737–41. doi: 10.1136/postgradmedj-2021-140195 37319159

[7] Dula J, Mulhanga A, Nhanombe A, Cumbi L, Júnior A, Gwatsvaira J, et al. COVID-19 Vaccine Acceptability and Its Determinants in Mozambique: An Online Survey. Vaccines. 2021;9(8):828. doi: 10.3390/vaccines9080828 34451953 PMC8402577

[8] Malik AA, McFadden SAM, Elharake J, Omer SB. Determinants of COVID-19 vaccine acceptance in the US. EClinicalMedicine. 2020;26:100495. doi: 10.1016/j.eclinm.2020.100495 32838242 PMC7423333

[9] Africa Centres for Disease Control and Prevention (CDC). Guidance On Expedited Regulatory Authorisation Process For Emergency Use of COVID-19 Vaccines In Africa. 2021;(January):18–9.

[10] WHO. Eight in 10 African countries to miss crucial COVID-19 vaccination goal. 2021.

[11] Biswas N, Mustapha T, Khubchandani J, Price JH. The Nature and Extent of COVID-19 Vaccination Hesitancy in Healthcare Workers. J Community Health [Internet]. 2021;(0123456789). Available from: doi: 10.1007/s10900-021-00984-3 33877534 PMC8056370

[12] Sevidzem Wirsiy F, Nkfusai NC, Ebot Ako-Arrey D, Kenfack Dongmo E, Titu Manjong F, Nambile Cumber S. Acceptability of COVID-19 Vaccine in Africa. Int J Matern Child Heal AIDS. 2021;10(1):134–8. 10.21106/ijma.482 PMC8039868 33868778

[13] Li M, Luo Y, Watson R, Zheng Y, Ren J, Tang J, et al. Healthcare workers’ (HCWs) attitudes and related factors towards COVID-19 vaccination: A rapid systematic review. Postgrad Med J. 2021;1–7. 37319159 10.1136/postgradmedj-2021-140195

[14] Luo C, Yang Y, Liu Y, Zheng D, Shao L, Jin J, et al. Intention to COVID-19 vaccination and associated factors among health care workers: A systematic review and meta-analysis of cross-sectional studies. Am J Infect Control [Internet]. 2021;000(April). Available from: doi: 10.1016/j.ajic.2021.06.020 34273461 PMC8278862

[15] Agyekum MW, Afrifa-Anane GF, Kyei-Arthur F, Addo B. Acceptability of COVID-19 vaccination among health care workers in Ghana. Adv Public Heal. 2021;2021:2021.03.11.21253374.

[16] CRD. CRD’s guidance for undertaking reviews in healthcare. New York: University of York NHS Centre for Reviews & Disseminatio. 2009. 1–277 p.

[17] Moher D, Liberati A, Tetzlaff J, Altman DG, Altman D, Antes G, et al. Preferred reporting items for systematic reviews and meta-analyses: The PRISMA statement. PLoS Med. 2009;6(7):e1000097. doi: 10.1371/journal.pmed.1000097 19621072 PMC2707599

[18] Ackah M, Yeboah CO, Ameyaw L. Risk factors for 30-day in-hospital mortality for in-patient with stroke in sub-Saharan Africa: Protocol for a systematic review and meta-analysis. BMJ Open. 2021;11(7):8–12. doi: 10.1136/bmjopen-2021-049927 34301662 PMC8311307

[19] Luchini C, Stubbs B, Solmi M, Veronese N. Assessing the quality of studies in meta-analyses: Advantages and limitations of the Newcastle Ottawa Scale. World J Meta-Analysis. 2017;5(4):80.

[20] Joseph L, Standen M, Paungmali A, Kuisma R, Sitilertpisan P, Pirunsan U. Prevalence of musculoskeletal pain among professional drivers: A systematic review. J Occup Health. 2020;62(1):e12150. doi: 10.1002/1348-9585.12150 32810918 PMC7434558

[21] Higgins JPT, Thompson SG. Quantifying heterogeneity in a meta-analysis. Stat Med. 2002;21(11):1539–58. doi: 10.1002/sim.1186 12111919

[22] Kabamba Nzaji M, Kabamba Ngombe L, Ngoie Mwamba G, Banza Ndala DB, Mbidi Miema J, Luhata Lungoyo C, et al. Acceptability of Vaccination Against COVID-19 Among Healthcare Workers in the Democratic Republic of the Congo. Pragmatic Obs Res. 2020;Volume 11:103–9. doi: 10.2147/POR.S271096 33154695 PMC7605960

[23] Fares S, Elmnyer MM, Mohamed SS, Elsayed R. COVID-19 Vaccination Perception and Attitude among Healthcare Workers in Egypt. J Prim Care Community Heal. 2021;12. doi: 10.1177/21501327211013303 33913365 PMC8111272

[24] El-sokkary RH, El Seifi OS, Hassan HM, Mortada EM, Hashem MK, Rabie M, et al. Predictors of COVID-19 vaccine hesitancy among Egyptian healthcare workers: a cross-sectional study. BMC Infect Dis. 2021;21:762. doi: 10.1186/s12879-021-06392-1 34353279 PMC8341553

[25] Adeniyi OV, Stead D, Singata-Madliki M, Batting J, Wright M, Jelliman E, et al. Acceptance of covid-19 vaccine among the healthcare workers in the eastern cape, south africa: A cross sectional study. Vaccines. 2021;9(6):1–11. doi: 10.3390/vaccines9060666 34207018 PMC8233726

[26] Elshora A, Abu-elenin MM. Acceptance of COVID-19 Vaccines Among Physicians in Mid Delta Region of Egypt: A Cross- Sectional Study. medRxiv Prepr. 2021; 10.1007/s11356-021-16574-8 PMC8504568 34636006

[27] Saied SM, Saied EM, Kabbash IA, Abdo SAEF. Vaccine hesitancy: Beliefs and barriers associated with COVID-19 vaccination among Egyptian medical students. J Med Virol. 2021;93(7):4280–91. doi: 10.1002/jmv.26910 33644891 PMC8013865

[28] Kanyike AM, Olum R, Kajjimu J, Ojilong D, Akech GM, Nassozi DR, et al. Acceptance of the coronavirus disease-2019 vaccine among medical students in Uganda. Trop Med Health. 2021;49(1):37. doi: 10.1186/s41182-021-00331-1 33985592 PMC8116637

[29] Ngasa NC, Ngasa SN, Armelle L, Tchouda S, Abanda C. Spirituality and other factors associated with COVID-19 Vaccine Acceptance amongst Healthcare Workers in Cameroon. priprint. 2021;1–16.

[30] Hussein AAM, Galal I, Makhlouf NA, Makhlouf HA, Abd-Elaal HK, Kholief KM, et al. A national survey of potential acceptance of COVID-19 vaccines in healthcare workers in Egypt. medRxiv [Internet]. 2021;(January):2021.01.11.21249324. Available from: https://www.medrxiv.org/content/10.1101/2021.01.11.21249324v1%0Ahttps://www.medrxiv.org/content/10.1101/2021.01.11.21249324v1.abstract

[31] Alle YF, Oumer KE. Attitude and associated factors of COVID-19 vaccine acceptance among health professionals in Debre Tabor Comprehensive Specialized Hospital, North Central Ethiopia; 2021: cross-sectional study. VirusDisease [Internet]. 2021;32(2):272–8. Available from: doi: 10.1007/s13337-021-00708-0 34222565 PMC8231083

[32] Guangul BA, Georgescu G, Azeze ZB. Healthcare workers attitude towards SARS-COVID-2. Glob J Infect Dis Clin Res. 2021;7:43–8.

[33] Ahmed MH, Kanfe SG, Jarso MH. Intention to receive vaccine against COVID-19 and associated factors among health professionals working at public hospitals in resource limited settings. PLoS One [Internet]. 2021;16(7 July):1–10. Available from: doi: 10.1371/journal.pone.0254391 34252143 PMC8274862

[34] Kweku Annan John Jude, Norman Betty Roberta, Mensah Boniface, Enimil Anthony, Kokuro Collins. Willingness to accept vaccination against SARS-cov-2: A survey of junior doctors. World J Adv Res Rev. 2021;9(3):159–66.

[35] Adejumo OA, Ogundele OA, Madubuko CR, Oluwafemi RO, Okoye OC, Okonkwo KC, et al. Perceptions of the COVID-19 vaccine and willingness to receive vaccination among health workers in Nigeria. Osong Public Heal Res Perspect. 2021;12(4):236–43. doi: 10.24171/j.phrp.2021.0023 34289295 PMC8408417

[36] Robinson E, Wilson P, Eleki B, Wonodi W. Knowledge, acceptance, and hesitancy of COVID-19 vaccine among health care workers in Nigeria. MGM J Med Sci. 2021;8(2):102.

[37] Oriji PC, Allagoa DO, Wagio TJ, Obagah L, Tekenah ES, Ozori SE. Hesitancy of Covid-19 Vaccination among Health Workers (other than Doctors) in a Tertiary Hospital in South-South, Nigeria. Asian J Res Infect Dis. 2021;7(1):21–31.

[38] Khairy A, Mahgoob E, Ahmed M. Acceptability of COVID-19 Vaccination among Healthcare Workers in Sudan: A Cross Sectional Survey. medRxiv. 2021; 10.26719/emhj.23.061 37306173

[39] Zammit N. Understanding hesitancy towards vaccination against SARS-COV2 among Health professionals in. priprint. 2021;

[40] Mudenda S, Mukosha M, Meyer JC, Fadare J, Godman B, Kampamba M, et al. Awareness and Acceptance of COVID-19 Vaccines among Pharmacy Students in Zambia: The Implications for Addressing Vaccine Hesitancy. priprint. 2021;1–22.

[41] Shekhar R, Sheikh AB, Upadhyay S, Singh M, Kottewar S, Mir H, et al. COVID-19 vaccine acceptance among health care workers in the united states. Vaccines. 2021;9(2):1–18. doi: 10.3390/vaccines9020119 33546165 PMC7913135

[42] Xu B, Gao X, Zhang X, Hu Y, Yang H, Zhou YH. Real-world acceptance of covid-19 vaccines among healthcare workers in perinatal medicine in China. Vaccines. 2021;9(7):1–10. doi: 10.3390/vaccines9070704 34199143 PMC8310137

[43] Gagneux-Brunon A, Detoc M, Bruel S, Tardy B, Rozaire O, Frappe P, et al. Intention to get vaccinations against COVID-19 in French healthcare workers during the first pandemic wave: a cross-sectional survey. J Hosp Infect [Internet]. 2021;108:168–73. Available from: doi: 10.1016/j.jhin.2020.11.020 33259883 PMC7699157

[44] Elharake JA, Galal B, Alqahtani SA, Kattan RF, Barry MA, Temsah MH, et al. COVID-19 Vaccine Acceptance among Health Care Workers in the Kingdom of Saudi Arabia. Int J Infect Dis [Internet]. 2021;109:286–93. Available from: doi: 10.1016/j.ijid.2021.07.004 34242765 PMC8260488

[45] Dzieciolowska S, Hamel D, Gadio S, Dionne M, Gagnon D, Robitaille L, et al. Covid-19 vaccine acceptance, hesitancy, and refusal among Canadian healthcare workers: A multicenter survey. Am J Infect Control. 2021;49:1152–7. doi: 10.1016/j.ajic.2021.04.079 33930516 PMC8079260

[46] Holzmann-Littig C, Braunisch MC, Kranke P, Popp M, Seeber C, Fichtner F, et al. Covid-19 vaccination acceptance and hesitancy among healthcare workers in Germany. Vaccines. 2021;9(7). doi: 10.3390/vaccines9070777 34358193 PMC8310090

[47] Abuown A, Ellis T, Miller J, Davidson R, Kachwala Q, Medeiros M, et al. COVID-19 vaccination intent among London healthcare workers. Occup Med (Chic Ill). 2021;71(4–5):211–4. doi: 10.1093/occmed/kqab057 34002797 PMC8194640

[48] Wonodi C, Obi-Jeff C, Adewumi F, Keluo-Udeke SC, Gur-Arie R, Krubiner C, et al. Conspiracy theories and misinformation about COVID-19 in Nigeria: Implications for vaccine demand generation communications. Vaccine [Internet]. 2022;40(13):2114–21. Available from: doi: 10.1016/j.vaccine.2022.02.005 35153088 PMC8830779

[49] Jegede AS. What led to the Nigerian boycott of the polio vaccination campaign? PLoS Med. 2007;4(3):417–22. 10.1371/journal.pmed.0040073 PMC1831725 17388657

[50] Gallè F, Sabella EA, Roma P, De Giglio O, Caggiano G, Tafuri S, et al. Knowledge and acceptance of COVID-19 vaccination among undergraduate students from central and southern Italy. Vaccines. 2021;9(6):1–13. doi: 10.3390/vaccines9060638 34200835 PMC8230551

[51] Lucia VC, Kelekar A, Afonso NM. COVID-19 vaccine hesitancy among medical students. J Public Health (Bangkok). 2020;1–5. 10.1093/pubmed/fdaa230 PMC7799040 33367857

[52] Tavolacci MP, Dechelotte P, Ladner J. Covid-19 vaccine acceptance, hesitancy, and resistancy among university students in france. Vaccines. 2021;9(6):1–13. 10.3390/vaccines9060654 PMC8232624 34203847

[53] Osei SA, Biney RP, Anning AS, Nortey LN, Ghartey-Kwansah G. Low incidence of COVID-19 case severity and mortality in Africa; Could malaria co-infection provide the missing link? BMC Infect Dis [Internet]. 2022;22(1):1–11. 35065613 10.1186/s12879-022-07064-4 PMC8783581

[54] Szmyd B, Karuga FF, Bartoszek A, Staniecka K, Siwecka N, Bartoszek A, et al. Attitude and behaviors towards sars-cov-2 vaccination among healthcareworkers: A cross-sectional study from Poland. Vaccines. 2021;9(3):1–14. 10.3390/vaccines9030218 PMC8000513 33806641

