# Supplementary material for: COVID-19 vaccine acceptance among health care workers in Africa: A systematic review and meta-analysis
Source: PLoS One. 2022 May 18;17(5):e0268711. doi: 10.1371/journal.pone.0268711 (PMC9116626; doi:10.1371/journal.pone.0268711)
Supplement: S2 Table — (DOCX) [file pone.0268711.s002.docx]

S2 Table: Search string for PubMed/Medline

| Search # | Search term |
| --- | --- |
|  | ‘’Covid-19’’ OR ‘’SARS Cov-2’’ OR 2019-nCov |
|  | vaccination OR immunization |
|  | ‘’acceptance rate’’ OR hesitancy OR ‘’Hesitancy rate’ |
|  | 1 AND 2 AND 3 |
|  | ‘’healthcare workers’’ OR HCW OR doctors OR Nurses OR Midwives OR Pharmacist OR Physiotherapist OR ‘’physical therapist’’ OR ‘’medical laboratory scientist’’ OR ‘’Occupational therapist’’ OR Dietician OR Dietitian OR Hospital |
|  | 4 AND 5 |
|  | Sub-Saharan Africa OR Africa OR ‘’Lower-middle income country’ ’OR LMIC |
|  | Algeria OR Angola OR Benin OR Botswana OR ‘’Burkina Faso’’ OR Burundi OR Cameroon OR ‘’Cape Verde’’ OR ‘’Central Africa Republic’ ’OR Chad OR Comoros OR Congo OR ‘’Democratic Republic Congo’’ OR Djibouti OR Egypt OR ‘’Equatorial Guinea’’ OR Swaziland OR Ethiopia OR Gabon OR Gambia OR Ghana OR Guinea OR Guinea-Bissau OR Ivory Coast OR Kenya OR Lesotho OR Liberia OR Libya OR Madagascar OR Malawi OR Mali OR Mauritius OR Mauritania OR Morocco OR Mozambique OR Namibia OR Niger OR Nigeria OR Rwanda OR ‘’Sao Tome and Principe’’ OR Senegal OR Seychelles OR ‘’Sierra Leone’’ OR Somalia OR ‘’South Africa’’ OR ‘’South Sudan’’ OR Sudan OR Tanzania OR Togo OR Tunisia OR Uganda OR Zambia OR Zimbabwe |
|  | 6 AND 7 OR 8 |
|  | Limit to January, 2020-date |
|  | Limit to Humans |
|  | Limit to English |
|  | 9 AND 10 AND 11 AND 12 |
