# Supplementary material for: COVID-19 vaccine acceptance among health care workers in Africa: A systematic review and meta-analysis
Source: PLoS One. 2022 May 18;17(5):e0268711. doi: 10.1371/journal.pone.0268711 (PMC9116626; doi:10.1371/journal.pone.0268711)
Supplement: S3 Table — (DOCX) [file pone.0268711.s003.docx]

**S3 Table**: Leave one out sensitivity analysis of pooled covid-19 acceptance rate among HCWs in Afica.

| **Author and Year** | Pooled estimate | 95% CI |
| --- | --- | --- |
| Nzaji et al, 2020 | 46 | 38-55 |
| Fares et al., 2021 | 47 | 38-55 |
| El-Sokkary et al.,2021 | 46 | 38-55 |
| Agyekum et al., 2021 | 46 | 37-55 |
| Dula et al., 2021 | 43 | 35-52 |
| Adeniyi et a., 2021 | 43 | 36-51 |
| Shehata et al.,2021 | 47 | 38-55 |
| Saied et al.,2021 | 46 | 37-55 |
| Kanyike et al., 2021 | 46 | 37-55 |
| Ngasa et al., 2021 | 46 | 36-55 |
| Aliae et al., 2021 | 46 | 36-55 |
| Alle et al.,2021 | 46 | 37-55 |
| Guangul et al., 2021 | 44 | 36-53 |
| Ahmed et al., 2021 | 46 | 37-55 |
| Annan et al., 2021 | 44 | 36-53 |
| Adejumo et al.,2021 | 45 | 36-54 |
| Robinson et al.,2021 | 45 | 36-54 |
| Oriji et al.,2021 | 46 | 37-55 |
| Khairy et al., 2021 | 45 | 36-54 |
| Zammit etal., 2021 | 45 | 36-54 |
| Mudenda et al.,2021 | 47 | 38-55 |
| Overall pooled estimate | 46 | 37-54 |
