# Supplementary material for: COVID-19 vaccine acceptance among health care workers in Africa: A systematic review and meta-analysis
Source: PLoS One. 2022 May 18;17(5):e0268711. doi: 10.1371/journal.pone.0268711 (PMC9116626; doi:10.1371/journal.pone.0268711)
Supplement: Attachment — Submitted filename: Specific Comments.pdf [file pone.0268711.s004.pdf]

Manuscript
Click here to access/download;Manuscript;manuscript.docx

COVID-19 Vaccine Acceptance among Health care workers in Africa: A Systematic Review and Meta-analysis

Martin Ackah[1,7*], Louise Ameyaw[2,7], Mohammed Gazali Saifu[3,7], Delali Pearl Afi Asubonteng[4] Cynthia Osei Yeboah[1], Eugene Narkotey Annor[5], Eunice Abena Kwartemaa Ankanpong[6], Hosea Boakye[8]

1. Department of Physiotherapy, Korle Bu Teaching Hospital, Accra, Ghana.
2. Department of Medicine, Achimota Hospital, Accra, Ghana.
3. Policy Planning Budgeting Monitoring and Evaluation Directorate, Ministry of Health, Accra, Ghana
4. Department of Physiotherapy, GA East Municipal Hospital, Accra, Ghana.
5. School of Public Health, East Tennessee State University, USA
6. Department of Occupational Therapy, School of Biomedical and Allied Health Science, University of Ghana
7. Department of Epidemiology, School of Public Health, College of Health Sciences, University of Ghana.
8. Department of Physiotherapy, LEKMA Hospital, Accra, Ghana

*Corresponding author

Email: martinackah10@gmail.com (MA)

Abstract

Introduction: Covid-19 vaccine acceptance, and hesitancy amongst Health Care Workers (HCWs) on the African continent have been examined through observational studies. However, there are currently no comprehensive reviews among these cadre of population in Africa. Hence, we aimed to review the acceptance rate and possible reasons for Covid-19 Vaccine non-acceptance/hesitancy amongst HCWs in Africa.

Methods: We searched Medline/PubMed, Google Scholar, and Africa Journal Online from 2020 to date. The Newcastle-Ottawa Quality Assessment tool adapted for cross-sectional studies was used to assess the quality of the retrieved studies. DerSimonian and Laird random-effects model was used to pool the covid-19 vaccine acceptance rate. Sub-group and sensitivity analyses were performed. Reasons for covid-19 vaccine hesitancy were also systematically analyzed.

Results: Twenty-one (21) studies were found to be eligible for review out of 513 initial records. The estimated pooled covid-19 vaccine acceptance rate was 46% [95% CI: 37%-54%]. The pooled estimated covid-19 acceptance rate was 37% [95% CI: 27%-47%] North Africa, 28% [95% CI: 20%-36%] in Central Africa, 48% [CI: 38%-58%] in West Africa, 49% [95% CI: 30%-69%] in East Africa, and 90% [CI: 85%-96%] in Southern Africa. The estimated pooled vaccine acceptance was 48% [95% CI:38%-57%] for healthcare workers, and 34% [95% CI:29%-39%] for the healthcare students. Major drivers and reasons were the side effects of the vaccine, vaccine's safety, efficacy and effectiveness, short duration of the clinical trials, covid-19 infections, limited information, and social trust.

Conclusion: The data revealed generally low acceptance of the vaccine amongst HCWs care workers across Africa. The side effects of the vaccine, vaccine's safety, efficacy and effectiveness, short duration of the clinical trials, covid-19 infections, limited information, and social trust were the major reasons for covid-19 hesitancy in Africa. The misconceptions and barriers to covid-19 vaccine acceptance amongst these cadre of professionals must be addressed as soon as possible in the continent to boost covid-19 vaccination rates in Africa.

Introduction

The current Covid-19 pandemic is a global public health emergency that offers significant challenges to health-care systems (1,2). ‘’Coronaviruses are large, enveloped, positive-strand RNA viruses that can be categorized into genera; alpha, beta, delta and gamma, of which alpha and beta are known to infect humans’’ (3). Human Corona Viruses (HCoVs) i.e. HCoV 229E, NL63, OC43 and HKU1 are endemic globally and account for 10%- 30% of upper respiratory tract infections in adults humans (3).

The current basic reproductive number (R0) of Severe Acute Respiratory Syndrome Coronavirus-2 (SARS COV-2) is estimated to be three and as a result the threshold of herd immunity for COVID-19 is roughly around 67 percent (4,5). This purport that after the population's acquired immunity reaches 67 percent and above, COVID-19 infection rates will start to decline (6).

Individual and community initiatives such as enhanced hand cleanliness, physical distancing, and the personal protective equipment are currently being used to reduce disease transmission. However, with the world facing an economic downturn and the future uncertain, a COVID-19 vaccine is perhaps the best option for halting the epidemic (7,8).

The SARS Cov-2 Development and Access Strategy established by Africa Center for Disease Control in 2020 aim to vaccinate at least 60% of African Population by 2022 to develop herd immunity (9). Africa has received approximately 143 million doses in total as of September, 2021, but only 39 million people, or around 3% of the continent's population, had been adequately vaccinated. In the United States, 52 percent of people are fully vaccinated, whereas in the European Union, 57 percent are(10). The willingness of HCWs to be vaccinated against COVID-19 acts as a valuable role model for the general public(11).

As the covid-19 vaccine becomes more widely available in Africa, Sevidzem Wirsty and colleagues(12) discussed and evaluated probable factors linked with vaccination acceptability in Africa as; ‘’Vaccine hesitancy, attitude and uptake by health care workers, misinformation, religion, immunization roll out plans, social influences and enabling environment’’(12). Vaccine aversion among the general public has a direct association to vaccine hesitancy among HCWs. Thus, HCWs role in vaccine acceptability cannot be underestimated as a result of their modeling behavior (13).

A rapid systematic review of global vaccine acceptance among Health Care Workers (HCWs) ranged from approximately 28% to 73% (6). Similarly, a comprehensive review and meta-analysis of cross-sectional studies of health workers' intentions to vaccinate against covid-19 indicated a moderate acceptance rate [i.e., 51 percent]. The authors did admit, however, that the population studied were largely from economically developed countries, which limited the study's generalizability(14). Clearly, this cannot be extended to represent HCW intentions to vaccinate against covid-19 in Africa.

Covid-19 vaccine acceptance, and hesitancy amongst HCWs on the African continent have been examined through observational studies.(7,15). However, there are currently no comprehensive reviews among these cadre of population in Africa. Hence, we aimed to systematically review the acceptance rate and possible reasons for Covid-19 Vaccine non-acceptance/hesitancy amongst HCWs in Africa. The outcome would enable stakeholders [i.e., policy makers, researchers and government) package effective health promotion measures to boost covid-19 vaccine uptake in Africa.

Specific objectives

1. To determine the level of Covid-19 Vaccine acceptance among HCWs in Africa
2. To assess the drivers of Covid-19 vaccine non-acceptance/hesitancy among HCWs in Africa.

Methods

Protocol registration and best practice

The Center for Reviews and Dissemination standards were followed in preparing this systematic review and meta-analysis(16). Also, the current review was conducted and reported according to the guidelines of the Preferred Reporting Items for Systematic Review and Meta-Analysis (PRISMA)(17) [see S1 Table]. The protocol was registered at PROSPERO: [CRD42021275065]

Eligibility criteria

Participants/Population

The participants were Health care workers as well as health science students from Africa continent, and adults HCWs (≥18 years] were included. HCW is operationally defined as; Doctors, Nurses, Pharmacists, allied health professionals, paramedics, and Healthcare students [i.e., medical students, nurse students etc.]. General population, other university students, and children were excluded.

Intervention/Exposure

The total number of health workers surveyed, number of health workers intending to receive vaccination against COVID-19, and COVID-19 vaccine acceptance rate were included. However, non-COVID-19 vaccine acceptance studies or covid-19 acceptance studies in the general population were excluded.

Types of studies

All primary studies such as longitudinal, cohort, case-control and cross-sectional studies reporting covid-19 vaccine acceptance and hesitancy among health care workers in Africa were included in the current review. Original observational studies published in English.

Studies reporting animal studies, reviews, commentaries, letter to editors. Covid-19 vaccine acceptance and hesitancy articles published in other language other than English. Covid-19 acceptability studies among health care workers outside the Africa continent [i.e., Asia, Europe, America, and Australia continents] were excluded.

Comparator

Not applicable

Setting

Africa

Outcome of interest

The outcome of interest was covid-19 vaccine acceptance/and hesitancy rate among HCWs in Africa. In addition, the reasons for covid-19 hesitancy were explored.

Information sources and Search strategies

Medline/PubMed, Google Scholar, Africa Journal Online, and MedRxiv (preprint) were searched. The search was restricted to studies published between January,2020 to date. The search was limited to articles published in English. Reference lists of articles that meet the inclusion and exclusion was checked to identify extra studies that were not captured by our electronic search.

Medical Sub-Heading (MeSH) terms and free text were used in the search approach. These terms were combined with the Boolean operators ‘OR' and ‘AND'. The key term includes; Covid-19, Vaccine, Hesitancy, acceptance, Health care worker, Africa, Sub-Saharan Africa. The full search string is shown in S2 Table.

Data screening and selection

The data screening and selection involved the following; Two co-authors [MA and LA] independently screened the titles and abstracts against the eligibility criteria. Full texts of the articles were then obtained against the inclusion and exclusion criteria. A disagreement was then resolved by consensus. To ensure that independent reviewers apply the selection criteria reliably, a screening guide was used(18).

Data extraction and management

MA and LA extracted the data from the eligible published articles using a pre-tested and standardized excel spreadsheet. Data such as the author's name, year of publication, country, survey period, study design, sample size, HCWs population, acceptance rate, questionnaire and, reasons for covid-19 acceptance/hesitancy rate were extracted. Mendeley was used to managed and remove duplicated articles.

Quality assessment and risk of bias

The Newcastle-Ottawa Quality Assessment tool adapted for cross-sectional studies (19) was used to assess the quality of the retrieved studies. It is graded on 10-point stars. This process was done by two independent reviewers and average was taken as a final score for that particular study. The Newcastle-Ottawa Quality Assessment tool contains three domains. Domain 1 evaluate the methodological quality of each study [5 stars], domain 2 assess the comparability of the study [2 stars] and domain 3 evaluate the outcome measure and related statistical analysis [3 stars] (19). Furthermore, the review rated the overall quality of the studies into three; [low risk of bias (7-10), moderate risk of bias (5-6), and high risk of bias (0-4)] (20).

Data synthesis

Extracted data was exported into Stata (version 16; Stata Cooperation, TX, USA) from Microsoft excel 2013 for all analyses. The characteristics of the studies were presented and summarized in a Table. The pooled vaccine acceptance among the HCWs was estimated using DerSimonian and Laird random-effects model (REM) at 95% confidence interval and presented in a forest plot. The presence of heterogeneity among studies was quantified by estimating variance using the I^2 statistics.

A sub-group analysis was performed based on sub-region (West Africa vs. East Africa vs. Southern Africa vs. North Africa) and type of participants (Healthcare workers vs. Healthcare students). Leave one out sensitivity analysis was performed to examine the effects of a single study on the overall pooled estimate. Publication bias was checked by the funnel plot and Egger's test

Results

The electronic search yielded 513 articles; 400 articles remained after the duplicates were deleted. After screening the abstracts and titles, 200 articles were eliminated. One hundred and seventy (170) were removed because they were unrelated to the current research. Thirty (30) full-text papers were evaluated for eligibility. Nine papers were eliminated from the final data synthesis, leaving only 21 articles. The results are display in Figure 1**.**

Characteristics of included study

Out of the 21 studies included, 7 were conducted in North Africa, 6 in West Africa, 6 in East Africa and an article each from central and southern Africa. The sample size ranged from 182 to 2133, totaling 14132 participants. The participants were mainly doctors, nurses, medical laboratory scientists, pharmacists, and allied health staff. The first survey was performed in March-April 2020, and the most recent was conducted in March-June 2021. The studies were all cross sectional and published between 2020 and 2021. Majority of the included had low-moderate risk of bias [20/21]. The findings are summarized in Table 1

Table 1: Characteristics of included studies [n= 14132]

Author and Year	Country	Participants	Survey period	Male (%)	Age/Years	Sample Size	Acceptance rate n (%)	Reasons for Vaccine Hesitancy	Quality assessment
Nzaji, 2020(21)	DR Congo	Doctors, Nurses, Midwives, and Laboratory Technicians	March/April, 2020	50.9	40.31±11.67, Majority; 25–40 (63%)	613	27.7	Not stated	Low
Fares, 2021(22)	Egypt	Doctors, Nurses, Pharmacists, Physiotherapists, and Dentists	Dec, 2020-Jan, 2021	18.7	Majority;17-35 (70.4%)	385	21	1. Lack of enough clinical trials, and<br>2. Fear of vaccine's side effects	Low
El-Sokkary ,2021(23)	Egypt	Doctors, Dentists, Pharmacist, and others	Jan, 2021	22.4	NA	308	26	1. Severity of Covid-19<br>2. Vaccine safety	Moderate
Agyekum, 2021(15)	Ghana	Doctors, Nurses/midwives, and Allied health	Jan/Feb, 2021	36.8	Majority; 30-39 (56.0%)	234	39.3	1. vaccine safety<br>2. vaccine side effects<br>3. Acquiring Covid-19 through vaccination	Moderate
Dula, 2021(7)	Mozambique		March, 2021	NA	NA	566	86.6	1. Vaccine side effects<br>2. Made to cause harm<br>3. Vaccine not effective	Moderate
Adeniyi, 2021(24)	South Africa	Doctors, Nurses, Pharmacists, Allied Health, Support staff	Nov/Dec, 2020	18.5	Majority; 26-55 (79.2%)	1308	90.1	Not stated	Low
Shehata, 2021(25)	Egypt	Doctors	March/June, 2021	40.6	Majority: 31-40 (71.5%)	1268	24.3	1. Vaccine side effects<br>2. Short duration of Clinical Trial<br>3. Concerns about safety and efficacy	High
Saied,2021(26)	Egypt	Healthcare Students	Jan, 2021	34.8	20.2±1.8	2133	34.9	1. Insufficient information about vaccine side effect<br>2. Insufficient information about the vaccine<br>3. Insufficient trust from vaccine source	Moderate
Kanyike, 2021(27)	Uganda	Healthcare Students	March, 2021	62.8	Majority: <25 (61.2%)	600	37.3	1. Vaccine side effects<br>2. Misinformation<br>3. ineffectiveness	Low
Ngasa, 2021(28)	Cameroon	Doctor, Nurse, laboratory technician, Pharmacist, Public health, student, Other	Not stated	51.8	29.1±6.6	371	45.4	1. Efficacy of the vaccine<br>2. Short duration of clinical trials<br>3. Adverse effects	Low
Aliae, 2021(29)	Egypt	Doctor, Nurse, laboratory technician, Pharmacist, student, Other	Dec, 2020-Jan, 2021	34.9	Majority; 18-45 (55.0%)	496	45.9	Not stated	Moderate
Alle,2021(30)	Ethiopia	Doctor, Anesthetists, Nurses, Midwives, Pharmacists, Laboratory Professional	Not stated	63.6	Majority; 18-25 (55.0%)	327	42.3	Not stated	Low
Guangul, 2021(31)	Ethiopia	Physicians, health officers, nurses, Lab Technicians, Pharmacist, others	Not stated	69.3	Majority; 18-29 (58.2%)	668	72.2	1. Concerns about safety,<br>2. Ineffective<br>3. Acquiring covid-19 through vaccination<br>4. Side effects,<br>5. Short duration of clinical trial	Low

Author and Year	Country	Participants	Survey period	Male (%)	Age/Years	Sample Size	Acceptance rate n (%)	Reasons for Vaccine Hesitancy	Quality assessment
Ahmed, 2021(32)	Ethiopia	All Health professionals	Jan-March, 2021	70.2	Majority;30-39 (54.0%)	409	33.2	Not stated	Low
Annan, 2021(33)	Ghana	Doctors	Not stated	49.2	Majority; 25-30 (83.0%)	305	66.9	Not stated	Low
Adejumo,2021(34)	Nigeria	Doctors, Nurse, Lab scientist, pharmacist, Physiotherapist, others	Oct, 2020	64.3	40.0±6.0, Majority 18-40 (72.9%)	1470	55.5	Not stated	Low
Robinson,2021(35)	Nigeria	All Health professionals	Dec, 2020-Jan, 2021	56.7	Majority; 30-49 (66.6%)	1094	48.8	1. Safety,<br>2. Ineffectiveness<br>3. Side effects<br>4. Fear of the unknown	Moderate
Oriji, 2021(36)	Nigeria	Nurses, Lab scientist, Pharmacist, others	April, 2021	25.3	Majority; <36 (47.8)	182	27.4	1. To see what will happen (fear)<br>2. Short duration of Clinical trial<br>3. Side effects<br>4. Safety issues<br>5. Lack of trust in government/manufacturer	Moderate
Khairy, 2021(37)	Sudan	Doctors, Nurse, Lab scientist, pharmacist, public health, others	March- April 2021,	46.7	35.3±10.6	576	57	Not stated	Low
Zammit, 2021(38)	Tunisia	Doctors, Nurses pharmacy, paramedic	Jan, 2021	26.6	37.4 ±9.5, Majority; <41 (66.1%)	493	48.1	Not stated	Low
Mudenda,2021(39)	Zambia	Pharmacy student	April, 2021	50.3	Majority; 18-29 (81.2%)	326	24.5	1. Side effects<br>2. Ineffectiveness<br>3. Safety issues<br>4. Short clinical trials	Moderate

Pooled covid-19 vaccine acceptance rate among HCWs in Africa.

The covid-19 vaccination acceptance rate was calculated using data from twenty-one (21) studies in Africa. Based on the DerSimonian and Laird random-effects model, meta-analysis revealed a pooled covid-19 acceptance rate of 46% [95% CI: 37%-54%] (Figure 2). However, there was significant variability among the studies [I^2=96%, p≤0.001].

Publication bias assessment

No evidence of publication bias was found after symmetrical inspection using the funnel plot (Figure 3) and egger's regression test (0.1654).

Sub-group and Sensitivity analysis for covid-19 vaccine acceptance rate

Sub-group analysis

As shown in Table 2, sub-group analysis was based on sub-regions (i.e., North Africa vs. Central Africa, East Africa vs. West Africa). The pooled estimated covid-19 acceptance rate was 37% [95% CI: 27%-47%] North Africa, 28% [95% CI: 20%-36%] in Central Africa, 48% [CI: 38%-58%] in West Africa, 49% [95% CI: 30%-69%] in East Africa, and 90% [CI: 85%-96%] in Southern Africa.

Similarly, further stratification by type of participants (health workers vs. health science students). The estimated pooled vaccine acceptance was 48% [95% CI:38%-57%] for health workers, and 34% [95% CI:29%-39%] for the healthcare students [Table 2]

Table 2: Data synthesis stratify by Sub-region and type of participants

Group	Number of datasets	Pooled estimate at 95%CI	I^2 [p-value]
**Sub-region**			
Central Africa	1	28% [95% CI: 20%-36%]	-
North Africa	7	37% [CI: 27%-47%]	92.47% [p≤0.001]
West Africa	6	48% [CI: 38%-58%]	87.31% [≤0.001]
South Africa	1	90% [CI: 85%-96%]	-
East Africa	6	49% [95% CI: 30%-69%]	96.50% [≤0.001]
**Type of participants**			
Health workers	18	48% [95% CI:38%-57%]	96.14% [≤0.001]
Health science student	3	34% [95% CI:29%-39%]	37.13% [≤0.001]

CI=Confidence Interval

Sensitivity analysis for covid-19 acceptance rate

Leave one out sensitivity analysis was performed to re-estimate the pooled effect on outcome of the remaining studies on the pooled covid-19 acceptance rate. The results revealed that, no single study had a signifcant impact on the overall result. The pooled acceptability rate of covid-19 vaccine ranged from 43% [95% CI:35%-52%] to 47% [95% CI: 38%-55%] (S3 Table)

Reasons for covid-19 vaccine non-acceptance among HCWs in Africa

The current systematic review and meta-analysis identified 8 main reasons for covid-19 vaccine hesitancy among HCWs in Africa. These includes; the side effects of the vaccine(7,15,22,25,27,28,31,35,36,39) the vaccine's safety (15,22,23,25,35,36,39), efficacy and effectiveness (7,25,27,28,31,35,39), short duration of the clinical trials (22,25,28,31,36,39), covid-19 infections (15,31), limited information (26,27), and social trust(26,36). The results are summarized in Table 3

Table 3: Reasons for Covid-19 vaccine hesitancy among HCWs in Africa

Reason	Number of studies	Citation
Side effects of the vaccine	10	(7,15,22,25,27,28,31,35,36,39)
Vaccine's safety	7	(15,22,23,25,35,36,39)
Efficacy and effectiveness	7	7,19–23,2
Short duration of the clinical trials	6	(22,25,28,31,36,39)
Covid-19 infections	2	(15,31)
Limited information	2	(26,27)
Social trust	2	(26,36)

Discussion

The systematic review, and meta-analysis was carried out to ascertained the covid-19 vaccine acceptance rate, and possible reasons for the vaccine's hesitancy amongst HCWs in Africa. The data revealed generally low acceptance of the vaccine amongst HCWs care workers across Africa, and considerable covid-19 vaccine reluctance. The possible reasons for the vaccine hesitancy were;

the side effects of the vaccine, concerns about the vaccine's safety, efficacy and effectiveness, short duration of the clinical trials, covid-19 infections, limited information, and social trust.

The overall acceptance rate for the covid-19 vaccination was 46% [95% CI: 37%-54%]. This is comparable to a previous systematic review and meta-analysis from the western world(14) and higher than a US observational based study(40). However, our estimate is lower than prior observational studies conducted in China(41), France(42), Saudi Arabia(43), canada(44), Germany(45) and United Kingdom(46). Low confidence in the vaccine, invasion of media misinformation, conspiracy theories, infodemic, religious beliefs, and possibly past vaccine hesitancy in the continent could all be factors contributing to the low covid-19 vaccination acceptance rate.

The study also revealed an estimated covid-19 vaccine acceptance rate of 34% [95% CI:29%-39%] by the healthcare students. The estimated value is lower than previous studies in Italy(47), US(48), and France(49). Complacency, exacerbated by low illness risk and low mortality in the continent since the epidemic began, could be contributing factors in this group of participants.

Certain common impediments to the acceptance of the vaccine seemed to be shared by HCWs across the continent. These included, side effects of vaccines, vaccine safety, efficacy and effectiveness of the vaccines, short duration of the clinical trials, the possibility of contracting Covid-19 infection from vaccines, limited information on the vaccines, and lack of social trust. Vaccine hesitancy is mostly induced by the dissemination of misleading information, primarily through social media platforms and with the assistance of anti-vaccination organizations(50). Biswas and colleagues conducted a scoping review analysis of 35 studies and found that HCWs worldwide have a 22.5% covid-19 reluctance rate on average. Their reasons for refusing the vaccination were identical to those revealed in this study (11).

In general, persuading individuals who are vaccine skeptics to change their beliefs is difficult, especially in a continent where there has been a history of vaccination resistance. Nevertheless, it's preferable to concentrate on disseminating positive and factual vaccine information while also strengthening healthcare workers' resistance to fraudulent information. Easy access and mandatory covid-19 vaccination policies in Africa is a good way to promote covid-19 immunization uptake. Encouragement of vaccine production within Africa, and comparison of these vaccines with others produced outside the continent, could build more confidence in the safety and efficacy of vaccines among health care workers in the continent. This would involve isolating local strains of the virus to be used in the production of vaccines and the conduction of clinical trials among locals. The outcome of this will be, more tailored interventions to the fight against the pandemic on the continent and will bring the fight against Covid-19 nearer home. This will also help debunk unfavorable theories about the intents behind the production of vaccines. Finally, the onus is on governments and significant international stakeholders in the pandemic fight to utilize social media to educate the public, especially HCWs, on facts concerning vaccines in order to help debunk some claims made by conspiracy theorists

This systematic review and meta-analysis have a number of limitations that should be acknowledged. First, the current review considered only English published papers and as a result some relevant articles maybe missed. Secondary, there was significant heterogeneity across the studies. Nevertheless, to the best of the authors’ knowledge, this is the first systematic review and meta-analysis on covid-19 acceptance and hesitancy rate in Africa. The review used well-validated systematic review and meta-analysis models that are fully compliant with current international standards and recommendations. Sensitivity analyses were performed to determine the robustness of the estimates obtained from the meta-analysis

Conclusion

The result of this review revealed generally low acceptance of the Covid-19 vaccine amongst HCWs care workers across Africa. Major drivers and reasons were the side effects of the vaccine, vaccine's safety, efficacy and effectiveness, short duration of the clinical trials, covid-19 infections, limited information, and social trust. The willingness of HCWs to be vaccinated against COVID-19 acts as a valuable role model for the general public and hence, the misconceptions and barriers to covid-19 vaccine acceptance amongst these cadre of professionals must be addressed as soon as possible in the continent

Acknowledgement

None

References

1. Zhou F. Clinical Course And Risk Factors For Mortality Of Adult In Patients With COVID-19 In Wuhan, China: A Retrospective Cohort Study. J Med Study Res. 2020;3(1):01–2.

2. Rieg S, von Cube M, Kalbhenn J, Utzolino S, Pernice K, Bechet L, et al. COVID-19 in-hospital mortality and mode of death in a dynamic and non-restricted tertiary care model in Germany. PLoS One [Internet]. 2020;15(11 November):1–16. Available from: http://dx.doi.org/10.1371/journal.pone.0242127

3. Paules CI. Coronavirus Infections — More Than Just the Common Cold. JAMA. 2020;323(8):707–8.

4. Randolph HE. Herd immunity: Understanding Covid-19 by Haley etal. Immunity. 2020;(January):19–21.

5. Salje H, Lefrancq N, Courtejoie N, Bosetti P, Paireau J, Andronico A, et al. Erratum: Estimating the burden of SARS-CoV-2 in France (Science DOI: 10.1126/science.abc3517). Science (80-). 2020;368(6498):208–11.

6. Li M, Luo Y, Watson R, Zheng Y, Ren J, Tang J, et al. Healthcare workers' (HCWs) attitudes and related factors towards COVID-19 vaccination: A rapid systematic review. Postgrad Med J. 2021;52:737–41.

7. Dula J, Mulhanga A, Nhanombe A, Cumbi L, Júnior A, Gwatsvaira J, et al. COVID-19 Vaccine Acceptability and Its Determinants in Mozambique: An Online Survey. Vaccines.

2021;9(8):828.

8. Malik AA, McFadden SAM, Elharake J, Omer SB. Determinants of COVID-19 vaccine acceptance in the US. EClinicalMedicine. 2020;26:100495.

9. Africa Centres for Disease Control and Prevention (CDC). Guidance On Expedited Regulatory Authorisation Process For Emergency Use of COVID-19 Vaccines In Africa. 2021;(January):18–9.

10. WHO. Eight in 10 African countries to miss crucial COVID-19 vaccination goal. 2021.

11. Biswas N, Mustapha T, Khubchandani J, Price JH. The Nature and Extent of COVID-19 Vaccination Hesitancy in Healthcare Workers. J Community Health [Internet]. 2021;(0123456789). Available from: https://doi.org/10.1007/s10900-021-00984-3

12. Sevidzem Wirsiy F, Nkfusai NC, Ebot Ako-Arrey D, Kenfack Dongmo E, Titu Manjong F, Nambile Cumber S. Acceptability of COVID-19 Vaccine in Africa. Int J Matern Child Heal AIDS. 2021;10(1):134–8.

13. Li M, Luo Y, Watson R, Zheng Y, Ren J, Tang J, et al. Healthcare workers' (HCWs) attitudes and related factors towards COVID-19 vaccination: A rapid systematic review. Postgrad Med J. 2021;1–7.

14. Luo C, Yang Y, Liu Y, Zheng D, Shao L, Jin J, et al. Intention to COVID-19 vaccination and associated factors among health care workers: A systematic review and meta-analysis of cross-sectional studies. Am J Infect Control [Internet]. 2021;000(April). Available from: https://doi.org/10.1016/j.ajic.2021.06.020

15. Agyekum MW, Afrifa-Anane GF, Kyei-Arthur F, Addo B. Acceptability of COVID-19

vaccination among health care workers in Ghana. Adv Public Heal. 2021;2021:2021.03.11.21253374.

16. CRD. CRD’s guidance for undertaking reviews in healthcare. New York: University of York NHS Centre for Reviews & Disseminatio. 2009. 1–277 p.

17. Moher D, Liberati A, Tetzlaff J, Altman DG, Altman D, Antes G, et al. Preferred reporting items for systematic reviews and meta-analyses: The PRISMA statement. PLoS Med. 2009;6(7):e1000097.

18. Ackah M, Yeboah CO, Ameyaw L. Risk factors for 30-day in-hospital mortality for in-patient with stroke in sub-Saharan Africa: Protocol for a systematic review and meta-analysis. BMJ Open. 2021;11(7):8–12.

19. Luchini C, Stubbs B, Solmi M, Veronese N. Assessing the quality of studies in meta-analyses: Advantages and limitations of the Newcastle Ottawa Scale. World J Meta-Analysis. 2017;5(4):80.

20. Joseph L, Standen M, Paungmali A, Kuisma R, Sitilertpisan P, Pirunsan U. Prevalence of musculoskeletal pain among professional drivers: A systematic review. J Occup Health. 2020;62(1):e12150.

21. Kabamba Nzaji M, Kabamba Ngombe L, Ngoie Mwamba G, Banza Ndala DB, Mbidi Miema J, Luhata Lungoyo C, et al. <p>Acceptability of Vaccination Against COVID-19 Among Healthcare Workers in the Democratic Republic of the Congo</p>. Pragmatic Obs Res. 2020;Volume 11:103–9.

22. Fares S, Elmnyer MM, Mohamed SS, Elsayed R. COVID-19 Vaccination Perception and

Attitude among Healthcare Workers in Egypt. J Prim Care Community Heal. 2021;12.

23. El-sokkary RH, Seifi OS El, Hassan HM, Mortada EM, Hashem MK, Rabie M, et al. Predictors of COVID-19 vaccine hesitancy among Egyptian healthcare workers : a cross-sectional study. BMC Infect Dis. 2021;21:762.

24. Adeniyi OV, Stead D, Singata-Madliki M, Batting J, Wright M, Jelliman E, et al. Acceptance of covid-19 vaccine among the healthcare workers in the eastern cape, south africa: A cross sectional study. Vaccines. 2021;9(6):1–11.

25. Elshora A, Abu-elenin MM. Acceptance of COVID-19 Vaccines Among Physicians in Mid Delta Region of Egypt : A Cross- Sectional Study. medRxiv Prepr. 2021;

26. Saied SM, Saied EM, Kabbash IA, Abdo SAEF. Vaccine hesitancy: Beliefs and barriers associated with COVID-19 vaccination among Egyptian medical students. J Med Virol. 2021;93(7):4280–91.

27. Kanyike AM, Olum R, Kajjimu J, Ojilong D, Akech GM, Nassozi DR, et al. Acceptance of the coronavirus disease-2019 vaccine among medical students in Uganda. Trop Med Health. 2021;49(1):37.

28. Ngasa NC, Ngasa SN, Armelle L, Tchouda S, Abanda C. Spirituality and other factors associated with COVID-19 Vaccine Acceptance amongst Healthcare Workers in Cameroon. priprint. 2021;1–16.

29. Hussein AAM, Galal I, Makhlouf NA, Makhlouf HA, Abd-Elaal HK, Kholief KM, et al. A national survey of potential acceptance of COVID-19 vaccines in healthcare workers in Egypt. medRxiv [Internet]. 2021;(January):2021.01.11.21249324. Available from:

https://www.medrxiv.org/content/10.1101/2021.01.11.21249324v1%0Ahttps://www.medrxiv.org/content/10.1101/2021.01.11.21249324v1.abstract

30. Alle YF, Oumer KE. Attitude and associated factors of COVID-19 vaccine acceptance among health professionals in Debre Tabor Comprehensive Specialized Hospital, North Central Ethiopia; 2021: cross-sectional study. VirusDisease [Internet]. 2021;32(2):272–8. Available from: https://doi.org/10.1007/s13337-021-00708-0

31. Guangul BA, Georgescu G, Azeze ZB. Healthcare workers attitude towards SARS-COVID-2. Glob J Infect Dis Clin Res. 2021;7:43–8.

32. Ahmed MH, Kanfe SG, Jarso MH. Intention to receive vaccine against COVID-19 and associated factors among health professionals working at public hospitals in resource limited settings. PLoS One [Internet]. 2021;16(7 July):1–10. Available from: http://dx.doi.org/10.1371/journal.pone.0254391

33. John Jude Kweku Annan, Betty Roberta Norman, Boniface Mensah, Anthony Enimil, Collins Kokuro. Willingness to accept vaccination against SARS-cov-2: A survey of junior doctors. World J Adv Res Rev. 2021;9(3):159–66.

34. Adejumo OA, Ogundele OA, Madubuko CR, Oluwafemi RO, Okoye OC, Okonkwo KC, et al. Perceptions of the COVID-19 vaccine and willingness to receive vaccination among health workers in Nigeria. Osong Public Heal Res Perspect. 2021;12(4):236–43.

35. Robinson E, Wilson P, Eleki B, Wonodi W. Knowledge, acceptance, and hesitancy of COVID-19 vaccine among health care workers in Nigeria. MGM J Med Sci. 2021;8(2):102.

36. Oriji PC, Allagoa DO, Wagio TJ, Obagah L, Tekenah ES, Ozori SE. Hesitancy of Covid-19 Vaccination among Health Workers (other than Doctors) in a Tertiary Hospital in South-South, Nigeria. Asian J Res Infect Dis. 2021;7(1):21–31.

37. Khairy A, Mahgoob E, Ahmed M. Acceptability of COVID-19 Vaccination among Healthcare Workers in Sudan : A Cross Sectional Survey. medRxiv. 2021;

38. Zammit N. Understanding hesitancy towards vaccination against SARS-COV2 among Health professionals in. priprint. 2021;

39. Mudenda S, Mukosha M, Meyer JC, Fadare J, Godman B, Kampamba M, et al. Awareness and Acceptance of COVID-19 Vaccines among Pharmacy Students in Zambia : The Implications for Addressing Vaccine Hesitancy. priprint. 2021;1–22.

40. Shekhar R, Sheikh AB, Upadhyay S, Singh M, Kottewar S, Mir H, et al. COVID-19 vaccine acceptance among health care workers in the united states. Vaccines. 2021;9(2):1–18.

41. Xu B, Gao X, Zhang X, Hu Y, Yang H, Zhou YH. Real-world acceptance of covid-19 vaccines among healthcare workers in perinatal medicine in China. Vaccines. 2021;9(7):1–10.

42. Gagneux-Brunon A, Detoc M, Bruel S, Tardy B, Rozaire O, Frappe P, et al. Intention to get vaccinations against COVID-19 in French healthcare workers during the first pandemic wave: a cross-sectional survey. J Hosp Infect [Internet]. 2021;108:168–73. Available from: https://doi.org/10.1016/j.jhin.2020.11.020

43. Elharake JA, Galal B, Alqahtani SA, Kattan RF, Barry MA, Temsah MH, et al. COVID-

19 Vaccine Acceptance among Health Care Workers in the Kingdom of Saudi Arabia. Int J Infect Dis [Internet]. 2021;109:286–93. Available from: https://doi.org/10.1016/j.ijid.2021.07.004

44. Dzieciolowska S, Hamel D, Gadio S, Dionne M, Gagnon D, Robitaille L, et al. Covid-19 vaccine acceptance, hesitancy, and refusal among Canadian healthcare workers: A multicenter survey. Am J Infect Control. 2021;49:1152–7.

45. Holzmann-Littig C, Braunisch MC, Kranke P, Popp M, Seeber C, Fichtner F, et al. Covid-19 vaccination acceptance and hesitancy among healthcare workers in Germany. Vaccines. 2021;9(7).

46. Abuown A, Ellis T, Miller J, Davidson R, Kachwala Q, Medeiros M, et al. COVID-19 vaccination intent among London healthcare workers. Occup Med (Chic Ill). 2021;71(4–5):211–4.

47. Gallè F, Sabella EA, Roma P, De Giglio O, Caggiano G, Tafuri S, et al. Knowledge and acceptance of COVID-19 vaccination among undergraduate students from central and southern Italy. Vaccines. 2021;9(6):1–13.

48. Lucia VC, Kelekar A, Afonso NM. COVID-19 vaccine hesitancy among medical students. J Public Health (Bangkok). 2020;1–5.

49. Tavolacci MP, Dechelotte P, Ladner J. Covid-19 vaccine acceptance, hesitancy, and resistancy among university students in france. Vaccines. 2021;9(6):1–13.

50. Szmyd B, Karuga FF, Bartoszek A, Staniecka K, Siwecka N, Bartoszek A, et al. Attitude and behaviors towards sars-cov-2 vaccination among healthcareworkers: A cross-sectional

study from Poland. Vaccines. 2021;9(3):1–14.

Supporting information Caption

S1 Table: PRISMA Checklist

S2 Table: Search strategy for the databases

S3 Table: Leave one out sensitivity analysis

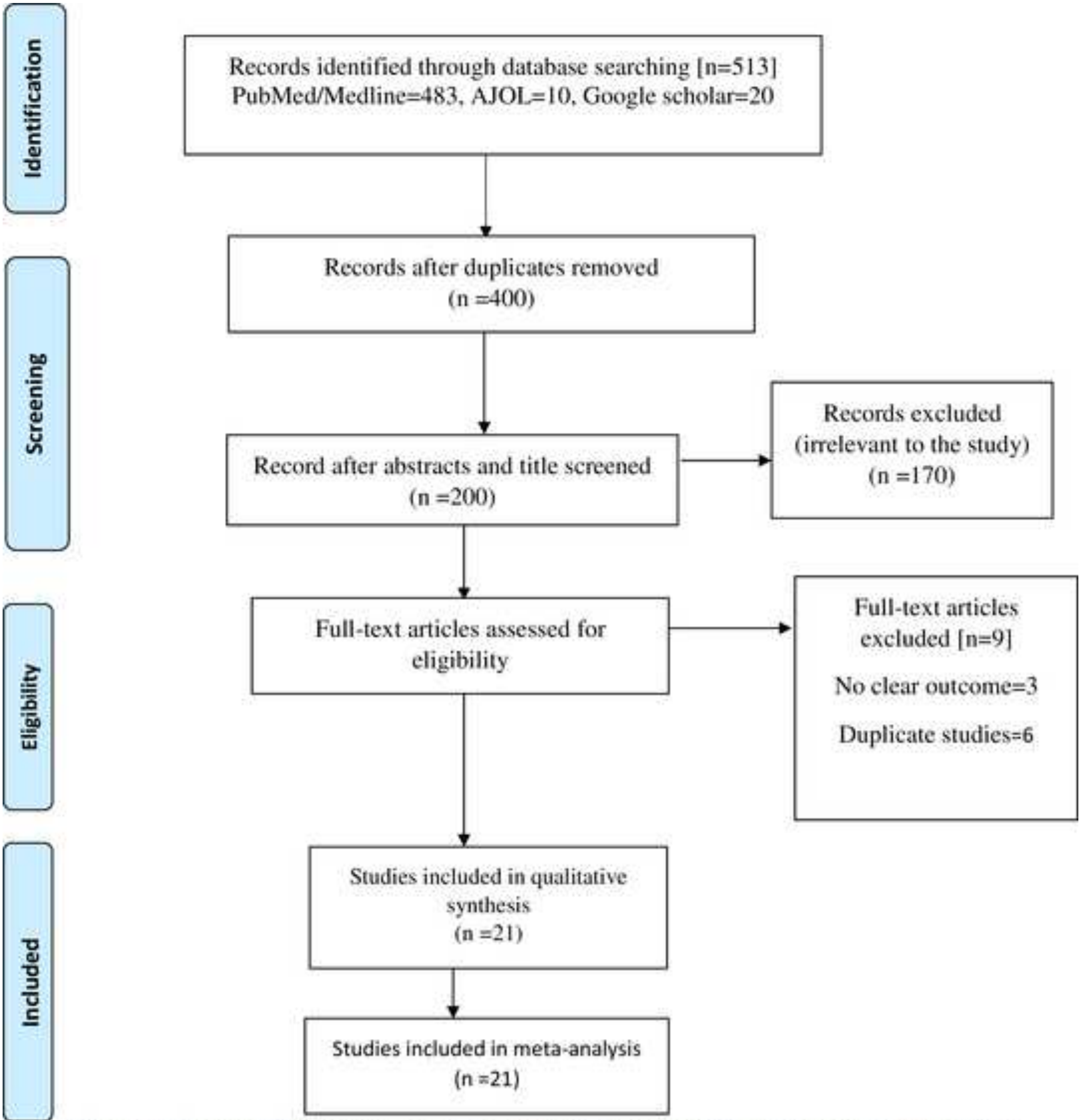

Figure 1: Preferred Reporting Items for Systematic Review and Meta-Analysis-Adapted flow showing the results of the search

Figure
Click here to access/download;Figure;Figure 2.tif

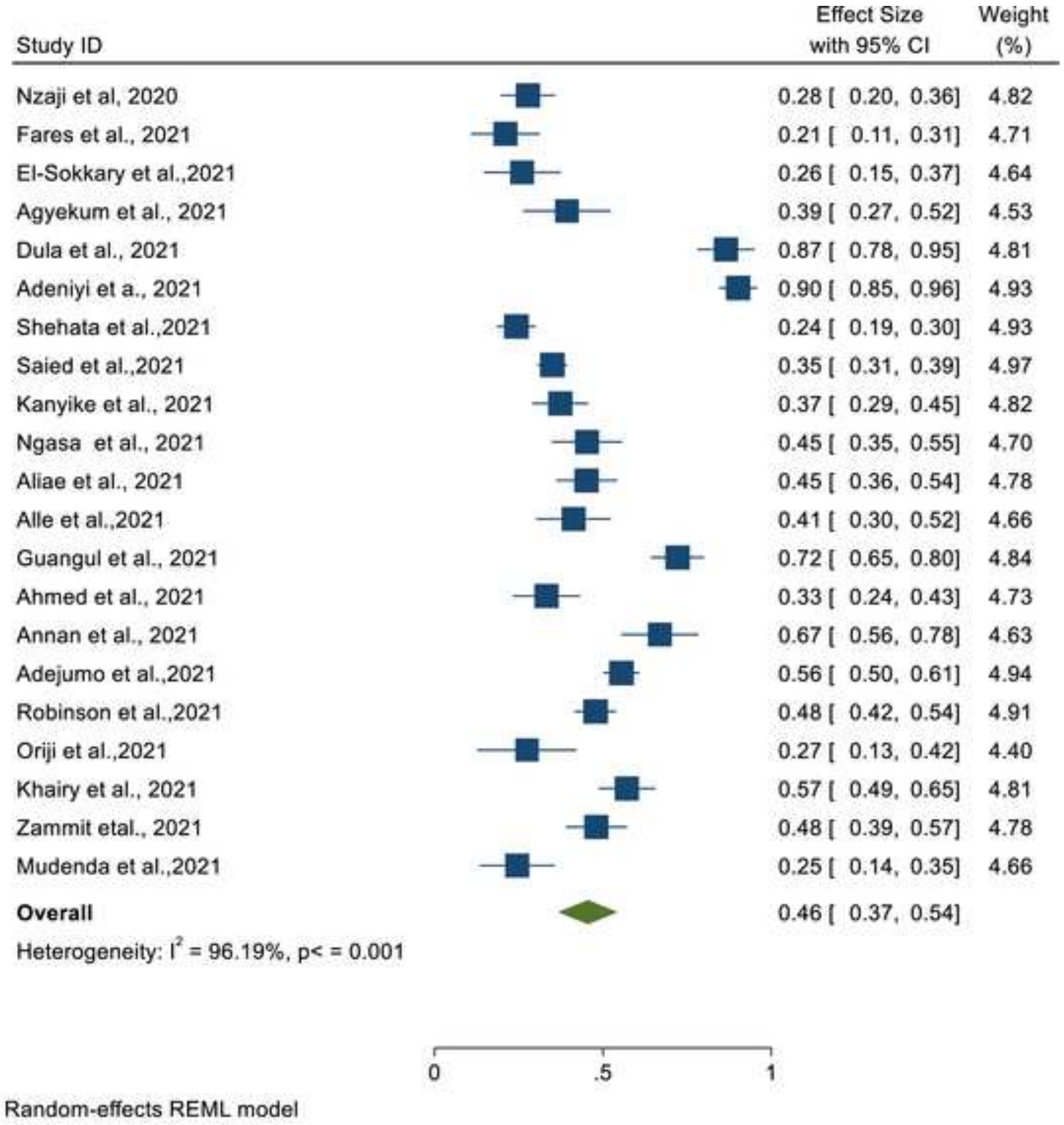

Figure 1: Covid -19 Vaccine acceptance rate among HCWs in Africa.

 Click here to access/download;Figure;Figure 3.tif

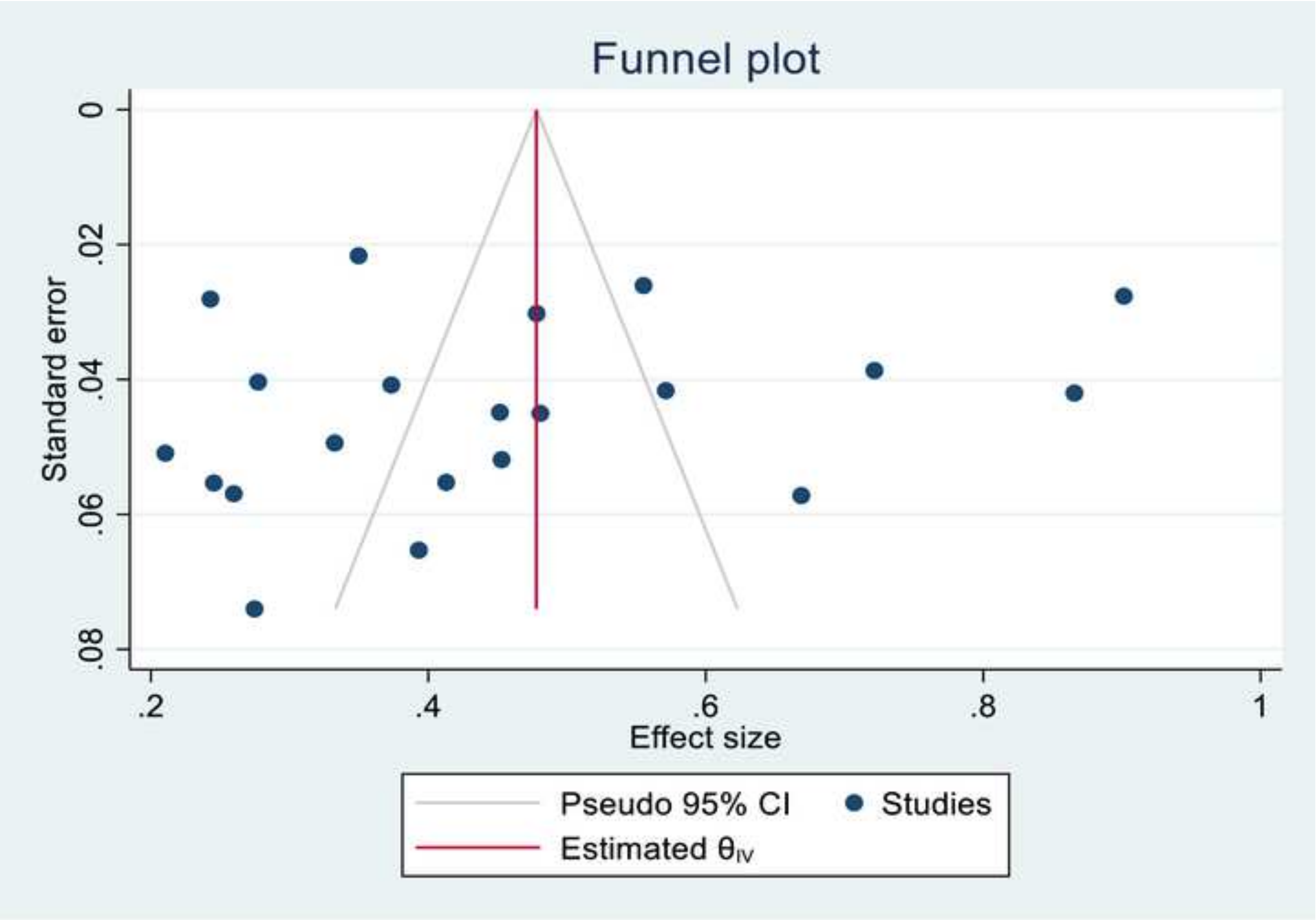

Figure 1: Assessment of publication bias

Click here to access/download
Supporting Information
S1 Table -Checklist.doc

Click here to access/download
Supporting Information
S 2 Table -Search strategies.docx

Click here to access/download
Supporting Information
S3 Table -senstivity analysis.docx
