# Supplementary material for: COVID-19 vaccine acceptance among health care workers in Africa: A systematic review and meta-analysis
Source: PLoS One. 2022 May 18;17(5):e0268711. doi: 10.1371/journal.pone.0268711 (PMC9116626; doi:10.1371/journal.pone.0268711)
Supplement: Attachment — Submitted filename: Response to reviewers.docx [file pone.0268711.s005.docx]

**Response to reviewers’ comments**

I sincerely express my warmest greetings to you and your prestigious journal for your comments and feedback. I write on behalf of my co-authors to submit our reply to your astute experienced reviewers' insightful comments. The methodology used follows a point-by-point approach to responding to all comments. Please see below for our response.

Reviewer 1

**Comment:** Grammar and spelling:
 I would recommend authors to review the grammar of the article. There are some sentences throughout the manuscript that are grammatically incorrect, the verb form does match the pronoun. There is also the use of pronouns that could be summarized differently, for example authors refer to MA and LA in the methods section, they could just refer to MA and LA as “two authors” or “two researchers. -COVID-19 should be capitalized. In many instances it is not capitalized, please correct.

Response: As recommended by the reviewer, the entire manuscript has been thoroughly read once again by all authors and a third independent editor to correct all grammatical errors which has improved the manuscript. Also, the authors have replaced the phrase ‘MA and LA’ with ‘’two authors’ ‘as suggested by the reviewer. Additionally, covid-19 is now capitalized

**Comment:** Page 5, first paragraph: I suggest authors to review the way they reference other studies. For example, on page 5, first paragraph; authors cite a study by Sevidzem Wirsty and colleagues. It is common to cite as “Sevidzem Wirsty et al.” I would also suggest to summarize information from other studies instead of adding the exact quote. For example, in the same paragraph, the phrase “Vaccine hesitancy, attitude and uptake by health care workers….” In the same paragraph the sentence “Vaccine aversion among the general public has a direct association to vaccine hesitancy among HCWs” does not have a reference, please provide reference.

Response: Authors have taken the reviewer’s comment into consideration and accordingly paraphrased and summarized the sentence. It now reads ‘’ Sevidzem et al identified and evaluated some probable link to vaccination acceptability in Africa. The factors included vaccination deployment plans, religious practices, vaccine hesitation, proliferation of misinformation, HCW attitudes towards the vaccine, social effects, and supportive environment (12)’. Additionally, ‘’Vaccine aversion among the general public has a direct association to vaccine hesitancy among HCWs’’ has now been referenced.

**Comment**: Please complete the protocol at PROSPERO. The protocol that is published does not match the one described in the submitted paper: Participants are different in the submitted manuscript and the PROSPERO protocol.

Response: Thanks for the comment. The PROSPERO protocol has been amended now.

**comment:** In the section “Information Sources and search strategies” on page 8: Please include the exact dates of the search (e.g., from January 2020 to Month/year).

Response: Thanks for the comment. The exact dates for the search have now been included i.e., January, 2020 September, 2021

**comment:** Figures and tables. The resolution of the figures is not high. I can see pixels in all figures, it would be desirable for the image to be clearer. All figures are named “Figure 1” please fix the numbers of the figures to match the text

**Response;** The authors thank the reviewer for these observations. The figures and table re-done for higher resolutions. All figures named ‘’Figure 1’’ were software and editorial issues. This will be rectified at the editorial level

**comment:** Comments in the discussion section: Page 16, 3rd paragraph: The authors claim the estimated value of acceptance rate they found is lower than those in Italy, US and France. I would suggest authors to write what the acceptance rates were in those country to have figures to compare to the African countries they included in the study.

Response: Authors have taken the reviewer’s comment into consideration and accordingly added the COVID-19 vaccine acceptance rate to those countries compared. The paragraph now reads’’ This is comparable to a previous systematic review and meta-analysis from the western world 51% (14) and higher than a US observational based study 36% (40). However, our estimate is lower than prior observational studies conducted in China 86.2% (41), France 76.9% (42), Saudi Arabia 64.9% (43), Canada 80.9% (44), Germany 91.7% (45) and United Kingdom 59% (46)’’……. The study also revealed an estimated COVID-19 vaccine acceptance rate of 34% [95% CI:29%-39%] by the healthcare students. The estimated value is lower than previous studies in Italy 91.9% (47), US 53% (48), and France 58% (49)

**Comment:** The manuscript could be improved by providing more information about the implications of the study regarding policy. The authors discuss policies such as easier access to vaccination and mandatory vaccination policies. They could provide information about what works for high vaccination acceptance in Africa, for example in Table 2, the acceptance of vaccination rate is 90%, significantly higher than other African countries. Authors could take the example of South Africa to discuss what works there versus other countries in Africa.

**Response:** Thanks for the comment. While there is no clear policy driving the high vaccination rate in south African on Policy implication, the authors have now discussed policy implication that could drive high vaccination acceptance in Africa, and probably other low- and middle-income countries and the global as a whole. it reads ‘’ it's preferable to concentrate on disseminating positive and factual vaccine information while also strengthening healthcare workers' resistance to fraudulent information. Easy access and mandatory COVID-19 vaccination policies in Africa is a good way to promote covid-19 immunization uptake. Encouragement of vaccine production within Africa, and comparison of these vaccines with others produced outside the continent, could build more confidence in the safety and efficacy of vaccines among health care workers in the continent. This would involve isolating local strains of the virus to be used in the production of vaccines and the conduction of clinical trials among locals. The outcome of this will be, more tailored interventions to the fight against the pandemic on the continent and will bring the fight against COVID-19 nearer home. This will also help debunk unfavorable theories about the intents behind the production of vaccines. Finally, the onus is on governments and significant international stakeholders in the pandemic fight to utilize social media to educate the public, especially HCWs, on facts concerning vaccines in order to help debunk some claims made by conspiracy theorist

Reviewer 2:

Comment: The paper is poorly written; specific issues are directly annotated to the pdf of the paper in the attached file. Moreover, the majority of the considered (statistical) approaches, such as the DerSimonian and Laird random-effects model (REM), the I^2 statistic, the funnel plot and the Egger test, are not adequately explained for non-expert readers. Instead, the leave-one-out meta-analysis is well-done enough.

**Response:** The authors would like to thank the reviewer for the annotated corrections. The grammatical errors, spacing, referencing, others have been duly corrected as highlighted in the current manuscript. Additionally, the considered statistical approaches have further been explained. It reads ‘’ Due to the presence of heterogeneity [ I^2^=96%, p≤0.001], a meta-analysis using the random effect model was used to pooled the vaccine acceptance rate among the HCWs at 95% confidence interval and presented in a forest plot. The presence of heterogeneity among studies was quantified by estimating the variance using the I^2^ statistics (21). The I^2^ takes values between 0 and 100%, and a value of 0% indicates absence of heterogeneity. I^2^ was interpreted based on Higgins and Thompson classification, percentages of 25%, 50% and 75% was considered as low, moderate and high heterogeneity, respectively’’
