# Supplementary material for: COVID-19 vaccine acceptance among health care workers in Africa: A systematic review and meta-analysis
Source: PLoS One. 2022 May 18;17(5):e0268711. doi: 10.1371/journal.pone.0268711 (PMC9116626; doi:10.1371/journal.pone.0268711)
Supplement: Attachment — Submitted filename: Response to reviewer.docx [file pone.0268711.s006.docx]

**Response to reviewers’ comments**

I sincerely express my warmest greetings to you and your prestigious journal for your comments and feedback. I write on behalf of my co-authors to submit our reply to your astute experienced reviewers' insightful comments. The methodology used follows a point-by-point approach to responding to all comments. Please see below for our response.

**Reviewer 1**

Comments: I thank the authors for their efforts in answering to comments. Some comments were partially addressed. For example, authors have not edited the word COVID throughout the manuscript (either in upper or lower case), some sentences still include grammatical mistakes throughout the manuscript. Re-reviewing the manuscript, I also realized that the inclusion and exclusion criteria for the manuscripts included in the review is poorly detailed.
Overall, I consider the research and results very important, but the manuscript lacks clarity in detailing methods and results and in other sections of the manuscript. The manuscript can improve enormously with a thorough review of grammar and reflection on the order of reporting methods and results

**Authors’ response:** The authors would like to use this opportunity to thank the reviewer for these vital observations**.** Authors have taken the reviewer’s comment into consideration and accordingly edited the word COVID in upper case throughout the manuscript. Moreover, the entire manuscript has been thoroughly read once again by all authors and a third independent editor to correct all grammatical errors which addresses the grammatical concerns raised by the reviewer. Also, the inclusion and exclusion criteria sections has been reviewed. Finally, the order of reporting methods and results follow a standard guidelines as reported in other reviews.
