# Supplementary material for: COVID-19 vaccine acceptance among health care workers in Africa: A systematic review and meta-analysis
Source: PLoS One. 2022 May 18;17(5):e0268711. doi: 10.1371/journal.pone.0268711 (PMC9116626; doi:10.1371/journal.pone.0268711)
Supplement: Attachment — Submitted filename: Response to reviewer.docx [file pone.0268711.s007.docx]

**Response to reviewers’ comments**

I sincerely express my warmest greetings to you and your prestigious journal for your comments and feedback. I write on behalf of my co-authors to submit our reply to your astute experienced reviewers' insightful comments. The methodology used follows a point-by-point approach to responding to all comments. Please see below for our response.

**Reviewer 1**

There are several points to address in the manuscript including grammatical changes, please see below:
**Comments regarding Introduction:**
**Comment**: In the Introduction, page 4, paragraph 3. Please correct the grammatical mistake from “…. with the world facing an economic downturn and the future uncertain,” to “…with the world facing an economic downturn and an uncertain future….”

**Response:** Thanks for the comment and suggestion. The grammatical error is now rectified as suggested.
**Comments regarding Methods:**
**Comment**: Authors list inclusion criteria for studies, the third criterion is “The total number of workers surveyed” but authors do not specify the total number that they considered appropriate for the inclusion criterion.

**Response:** Thanks for the comment. The third criterion is now deleted to prevent further ambiguity. It is very important to note that the removal of this criterion does not affect the included studies in this review in anyway.

**Comment**: In the section “Exclusion criteria”, authors mention they exclude studies focused on the general population twice, in exclusion criterion 1 and in exclusion criterion 2, please remove redundancy.

**Response:** Thanks for the comment and observation. Redundancy is checked and removed as suggested**.**
**Comment**: In section “Information sources and search strategies”, please correct the sentence “Reference lists of articles that met the inclusion and exclusion were manually checked to identify extra studies….” to “Reference lists of articles that met the inclusion and exclusion criteria were reviewed manually to identify additional studies……”

**Response:** Thanks for the comment and suggestion. The sentence is now rectified as suggested.
**Comment**: The study has two objectives: to determine the level of COVID-19 vaccine acceptance and to assess the drivers of COVID-19 vaccine non-acceptance/hesitancy. However, authors do not specify how they systematically assessed drivers of COVID-19 vaccine non-acceptance.

**Response:** Thanks for the comment and the observation. We have made a statement to that effect under the data synthesis section. It reads ‘’ The drivers/factors for COVID-19 vaccine non-acceptance/hesitancy among HCWs in Africa were systematically reviewed. A factor/driver for COVID-19 vaccine non-acceptance/hesitancy was eligible if it had been assessed and data from at least two studies were available’’.

**Comments regarding the Results section:**
**Comment**: Additionally, authors should clarify what is social trust, how is social trust defined in the studies they reviewed. Reasons for hesitancy are pretty clear (e.g. safety, effectiveness, duration of clinical trials), except for the concept of social trust. Please clarify in results and discussion.

**Response:** Thanks for the comment. Concept of social trust is now defined and clarified in the results and discussion. It’s defined as ‘’ insufficient trust in the vaccine’s source, lack of trust from the manufacturers, lack of trust from governments’’.
**comment:** In the results section, a subtitle needs to be changed from “Characteristics of included study” to “Characteristics of the studies”.

**Response:** Thanks for the comment and suggestion. The subtitle is now rectified as suggested.

**Comment:** Table 2 needs a proper legend. The legend does not specify what the pooled estimate is. A table needs to be able to stand by itself, here the readers do not know if the pooled estimate is the percentage of acceptance or the percentage of hesitancy.

**Response:** Thanks for the comment. The Table 2 legend is rectified elaboratively.
**Comments regarding the Discussion section:**
**Comment:** In the discussion section, second paragraph, authors mention factors that contribute to vaccine hesitancy (media misinformation, conspiracy theories etc). Authors do not have a reference for these factors. Did they find these factors in the studies they analyzed? Please add reference or clarify.

**Response:** Thanks for the comment. The authors have referenced the statement appropriately.
**Comment:** In the discussion section, third paragraph, authors also make a conjecture about why healthcare students show low acceptance rates, but authors do not cite where this information comes from. Was this found in the studies they analyzed? Did they do a review of media articles to make these conjectures? Please add appropriate reference

**Response:** Thanks for the comment. The authors have referenced the statement appropriately.
